# Supplementary material for: Inhibition of ATR Reverses a Mitochondrial Respiratory Insufficiency
Source: Cells. 2022 May 24;11(11):1731. doi: 10.3390/cells11111731 (PMC9179431; doi:10.3390/cells11111731)
Supplement: Supplementary file 1 [file cells-11-01731-s001.zip › cells-1721133-proofed-supplementary/cells-1721133-proofed-supplementary s1.pdf]

SUPPLEMENTAL METHODS

Nematode Strains and Maintenance. Full genotypic information for all strains used in this study is provided in **Table S4**. Lines were maintained at 20˚C on standard NGM agar plates seeded with *E. coli* (OP50) [33]. *atm-1(gk186)* mutants are homozygous viable and contain a 548 bp deletion that results in two aberrant *atm-1* transcripts, both of which encode non-functional proteins [82]. *atl-1(tm853)* mutants are maternal-effect lethal, meaning only homozygous *atl-1(tm853)* progeny derived from *atl-1(tm853)*/+ heterozygotes are viable. The *atl-1(tm853)* mutation contains a 720 bp deletion in the *atl-1* locus that results in no detectable ATL-1 protein by western analysis [48]. Although technically not necessary for the *atm-1(gk186)* mutation, we chose to place both mutations in the *nT1(IV:V)* reciprocal chromosomal translocation background. We also generated two independent *atm-1(gk186); atl-1(tm853)* double mutant *nT1* lines. As well, we constructed and maintained a control *nT1* line that contained no additional mutations and re-generated the wild type genotype upon loss of the *nT1* chromosomal pair. This approach of equally using *nT1* to carry all test genotypes, whether required or not, circumvented concerns of a possible untoward maternal effect caused by the *nT1* translocation itself.

Bacterial Feeding RNAi. All bacterial feeding RNAi constructs were designed in the pL4440 vector backbone and maintained in HT115 bacteria [34]. RNAi clones targeting *atp-3, isp-1, nuo-6* and *skn-1* have been described previously, along with the procedure for feeding RNAi dilution [13,44]. RNAi targeting *atl-1* was constructed using the following PCR primers and wild type (N2) genomic DNA as template: Forward primer: 5'-CTGTCTAAGCTTATTGAACGGCTGTCTGAATGTGC-3', Reverse primer: 5'-GATCTCTCGAGCGATCGGGCAAATGACAAGATTCC-3'. Feeding RNAi constructs used for the DDR screening study were obtained from the Ahringer RNAi library [83]. For assays comparing growth rate and life span across strains (**Figs. 1** and **2**, main text), eggs of the same chronological age from all strains were simultaneously seeded onto RNAi lawns that were prepared in unison to control for RNAi batch effects.

RNAi efficacy in *ncl-1* mutants was tested as follows: Worms were fed bacteria containing increasing amounts of *skn-1* RNAi from the arrested L1 stage onward. At the L4/YA boundary, exactly 20 animals were transferred to fresh *skn-1* RNAi plates and then allowed to lay eggs for another 24 hours. The number of dead eggs and hatchlings on each plate was then counted (~1000 progeny per plate).

Lifespan Analyses. Lifespan studies were performed as described previously [84], without the use of FUdR. All plates were maintained at 20ºC. The Log-rank test was used to analyze the effect of gene knock-out or RNAi knockdown. The first day of adulthood was designated as day one unless otherwise noted. Animals that bagged, desiccated or suffered gonad extrusion were censored. Such animals were included in life-span analyses up to the point of censorship and were weighted by half in mortality calculations.

Microscopy. Images of fluorescent worms were captured using an Olympus SZX16 fluorescence microscope connected to an Olympus DP71 CCD camera. Worms were immobilized for imaging by transferring animals to plates at 4ºC. Total worm fluorescence was quantified using ImageJ [85]. All images were collected on the first day of adulthood unless otherwise specified. For strains containing the *Phsp-6::GFP* or *Ptbb-6::GFP* reporter gene, background fluorescence in the head was excluded from signal integration. Six to 10 worms were quantified for each tested condition. For worm length measurements, the Segmented Line Function of ImageJ was used in conjunction with worm DIC images [85]. Up to 10 worms were quantified for each tested condition. For confocal imaging, worms containing the HXK2::GFP mitochondrial reporter were mounted on 5% agarose pads in 20 μL of 1 mM levamisole and fluorescent images collected using a Zeiss LSM 780 confocal microscope. In **Fig. 7C**, to determine whether differences existed in the morphology of mitochondria between the four tested conditions, four randomly selected images from each condition were mixed and then grouped by a scorer who was blind to image identity. In a set of 16 images, 10 were co-grouped correctly. The probability that this grouping occurred by chance

was modeled using an Excel VBA macro that randomly binned the images into four groups of four, 10,000 times (**File S2**). The final *p-value* was <0.0024 and deemed significant.

mRNA and mtDNA Quantitation. For quantitation of mRNA, 1000 first-day adult worms were washed in S-Basal [33], flash frozen in liquid nitrogen, then RNA extracted with an RNeasy kit (Qiagen). cDNA was generated using a High Capacity cDNA Reverse Transcription kit (Applied Biosystems). Quantitative PCR was performed using a 7500 Real Time PCR (Applied Biosystems) in conjunction with RT^2 SYBR Green/ROX reagent (Qiagen). Fold change in each mRNA of interest was determined using the ΔΔCt method normalized to the geometric mean of *cdc-42*, *pmp-3*, and *Y45F10D.4* [35]. For quantitation of mitochondrial DNA (mtDNA), PCR primer pairs targeting mtDNA-encoded *ctb-1* and intron 4 of genomic DNA-encoded *ama-1* were synthesized and used as described [36]. The ratio of mitochondrial DNA to nuclear DNA was then calculated using Real Time PCR, using *ama-1* as the normalizing quantity [37]. A complete list of qPCR primers is provided in **Table S5**.

Mutation Screening Assays:

Unc Reversion Assays: We used the frequency of Unc phenotypic reversion in *unc-93(e1500)* or *unc-58(e665)* mutants exposed to mitochondrial ETC disruption as one measure of nuclear DNA mutation rate. For both genotypes, 50 L1 larvae were seeded individually onto 10 cm bacterial feeding RNAi lawns targeting *atp-3* (1/20th strength); *rpa-1* (/10th strength) or vector-control. Once each worm population had starved, animals were collected in S-basal then transferred to a 2% peptone-enriched NGM-agar plate (10cm) seeded with wild type *E. coli* (strain RW2) and allowed to starve again. This step was repeated a second time. By this stage, plates containing revertants were easily identifiable and each such plate was scored as a single mutagenic event. Significance testing was undertaken using a Fisher's exact test, comparing the number of

lethal mutations detected in the *atp-3* population against vector control. Significance was set at $p<0.05$. *rpa-1* treated worms served as a positive control.

<u>Lethal Mutation Assays:</u> To score nuclear DNA mutation rate, 50 worms containing the *eT1(III;V)* (strain BC2200, **Table S4**) or the *nT1(IV;V)* (strains TJ5013 and SLR004) reciprocal chromosomal translocations were cultured from the L1 larval stage on bacterial feeding RNAi lawns targeting *atp-3* (across a range of dilutions) or vector control. Four hundred F1 progeny containing *eT1* or *nT1* were subsequently collected and then individually singled onto 6cm NGM/OP50 plates. Absence of Dpy Unc (BC2200) or wild type (TJ5013, SLR004) F2 progeny indicated the presence of a balanced, lethal mutation event in the F1 parent. Significance testing was undertaken using a Fisher's exact test, comparing number of lethal mutations detected in each *atp-3* population against vector control. Significance was set at $p<0.05$.

<u>Lac-Z Frameshift Assay:</u> Strain NL3400 contains the P*hsp-16.2::ATG(A)*$_{17}$*::GFP::LacZ* out-of-frame reporter gene. Mutations that disrupt the poly-(A) leader sequence and bring the reporter gene into frame provide a quantitative assessment of nuclear DNA mutation rate. Strain NL3401 contains an out-of-frame reporter gene identical to that in NL3400 worms except for the absence of the 17 nucleotide adenine repeat. Differences in the mutation frequency between NL3400 and NL3401 indicate differences in the types of DNA damage mediating reporter frameshifting. To measure nuclear DNA mutation frequency, fifty NL3400 worms were cultivated for one generation on bacterial feeding RNAi targeting *atp-3* (1/20th strength); *rpa-1* (/10th strength) or vector-control. Following a one hour heat shock, β-galactosidase activity was measured [86]. This assay, we discovered, was confounded by the presence of functional β-galactosidase in the HT115 feeding RNAi bacteria and provided no reliable results, which is contrary to previous reports suggesting its successful use under such conditions [86]. Examples of our results are provided in **Supplemental Fig. S2B**. Disruption of DNA damage response genes often results in severe

pathology and in worms the subsequent tissue invasion by their bacterial food source may have been responsible for many of the apparent positive results in the original screens in which both reporter genes were developed and employed.

<u>*TUNEL Assay*</u>: The 'terminal deoxynucleotidyl transferase dUTP nick end labeling' (TUNEL) assay was used to measure levels of ssDNA and dsDNA breaks in *isp-1(qm150)* mutants relative to wild type (N2) worms. Briefly, ~1000 animals were fixed in paraffin, sectioned, then stained for DNA breaks using the PromoKine Colorimetric DNA Fragmentation Detection Kit (IHC) (PK-CA577-K403) which utilizes Br-dUTP, a biotin-labeled anti-BrdU antibody, and an HRP-streptavidin conjugate to detect DNA lesions. For fixation and sectioning, washed worms (S-Basal) were immersed in 4% paraformaldehyde for 16 hr at 4℃ then dehydrated using increasing concentrations of ethanol (70%, 80%, 95%, 100%) then xylene. Worms were subsequently embedded in paraffin and sectioned (3μm) using a Leica RM2125 microtome. Sections were serially rehydrated in ethanol (100%, 90%, 80%, 70%) and washed in phosphate buffered saline, then the tissue permeabilized with proteinase K. Sections were processed for TUNEL using the DNA Fragmentation Detection Kit and according to the manufacturer's instructions. Nuclei were counterstained with DAPI. Staining was imaged by bright field and fluorescence microscopy (Zeiss Axioskop 2). Controls included γ-irradiated N2 worms, mutant *nuc-1(e1392)* worms (1.4 Gy/min, 16hrs), as well as worms treated with elevated NaCl (350mM), which purportedly results in DNA strand breakage [49]. *nuc-1* encodes a DNase II homolog and is required for DNA degradation during apoptosis [87].

Nucleotide Extraction and Mass Spectrometry. Quantitation of deoxyribonucleotide and ribonucleotide species in whole-worm extracts of *isp-1(qm150)* and wild-type (N2) animals was undertaken using HPLC-MS. Briefly, 30,000 stage-synchronized worms were collected and washed in cold S-Basal [33]. Worms were resuspended in HPLC-grade water, then homogenized with a Balch homogenizer, as described previously

[88]. Nucleotides were extracted using 6% TCA and water-saturated diethyl ether, following the procedure of Huang and colleagues [89]. Sample pH was neutralized with saturated $NaHCO_3$. LC-MS analyses were conducted using a Thermo Fisher/Dionex Ultimate 3000 HPLC connected in-line with a Thermo Fisher Q Exactive mass spectrometer. HPLC conditions were: Waters XTerra-MS C18 column (3.5 μm, 150 mm x 2.1 mm i.d.); mobile phase A – 5 mM Hexylamine and 0.5% diethylamine in water, pH 10; mobile phase B – 50% acetonitrile in water; flow rate – 400 μL/min; gradient – 1% B to 20% B over 10 minutes followed by 20% B to 30% B over 10 minutes. Full scan mass spectra were acquired on the Orbitrap using negative ion detection over a range of m/z 300 – 800 at 70,000 resolution (m/z 300). Nucleotide identification was based on accurate mass (± 5 ppm) and agreement with the HPLC retention time of authentic standards. Quantification was accomplished by integration of extracted ion chromatograms of each metabolite, followed by comparison with corresponding standard curves. Six nucleotides fell below our detection limit using this approach (dGMP, dGDP, dGTP, GMP and the cyclic nucleotides cAMP and cGMP). Tests for significance were undertaken using the programs Graphpad and Q-value as follows: Nucleotides that differed significantly between strains, or within strain but between larval stages, were identified using *Student's t-test* (significance defined as $p<0.05$). Results were then adjusted for multiple comparisons using the False Discovery Rate approach (5% FDR, equal variance assumption). To determine whether nucleotide pool sizes differed between strains, mean values of relevant nucleotide species were summed and their individual standard errors were used to calculate a nucleotide pool standard error using routine error propagation techniques.

R-loop Quantification. To quantify R-loop formation in worms exposed to mitochondrial ETC disruption, we used an antibody selective for DNA::RNA hybrids (αS9.6, Kerafast) [38]. Briefly, 5000 synchronized worms were cultured on bacterial feeding RNAi targeting *isp-1* (1/10th strength), *atl-1* (9/10th strength), both genes combined (1/10th strength *isp-1* + 9/10th strength *atl-1*), or vector control, from the L1 larval stage to

the first day of adulthood. Animals were collected in S-Basal, washed thrice in the same medium to remove eggs and bacteria, then flash frozen in liquid N_2. Genomic DNA (gDNA) was subsequently extracted using a DNeasy kit (Qiagen), and both the proteinase K and RNase A steps were included in the purification protocol, according to the manufacturers instructions. 250 ng of gDNA from each sample was next transferred to a Hybond H+ Nylon membrane (GE Healthcare) using a Hybri-Slot manifold (BRL). The DNA was UV-crosslinked to the membrane (1200 Joules, using a Stratagene Stratalinker 1800), then blocked in 5% fat-free milk powder dissolved in TBS-T (Tris-buffered saline + 0.1% Tween-20, abbreviated 5% milk powder (MP)). DNA::RNA hybrids were detected using αS9.6 primary antibody (Kerafast, 1:2000 in 5% MP, 16 hours, 4˚C), goat α-mouse secondary antibody (1 hour, Santa Cruz Biologicals, 1:2000 in TBS-T) and ECL (GE Healthcare). Concurrently, identical gDNA samples were run on a 1% agarose gel to separate gDNA by size. This gel was southern-blotted to a Hybond H+ Nylon membrane then incubated with αS9.6 primary antibody, as described above. All resulting data was quantified using ImageJ. ECL images were digitized with an HP8610 scanner at 600 dpi resolution. Three fully independent experimental replicates were collected. Significance testing was undertaken using Student's t-test, with $p<0.05$ considered statistically significant.

Spliceosome Reporter Assays. To test if worms exposed to mitochondrial ETC disruption have reduced mRNA splicing efficiency, we utilized the *egl-15* and *ret-1* alternate splicing reporter genes engineered by Kuroyanagi and colleagues [39,40] (strains KH1125, KH2283, KH928, **Table S1**). Briefly, the *egl-15* reporter gene fluoresces green when exon 5B is incorporated but red in its absence. The *ret-1* splicing reporter is comprised of a pair of synthetic genes encoding different fluorescence reporter proteins that have been frame-shifted relative to each other such that they are sensitive to the presence or absence of exon 5 for their correct translation. Synchronized eggs from each reporter strain were placed on relevant bacterial feeding RNAi lawns then cultured until day one of adulthood (20˚C). Worms were immediately imaged

using an Olympus SZX16 fluorescence dissecting microscope connected to an Olympus DP71 CCD camera, using filters for GFP and RFP/mCherry.

Nematode Oxygen Consumption:

Nematode oxygen consumption measurements using a Seahorse XFe24 Analyzer were undertaken as follows: Synchronized N2 worm populations were cultured on HT115 bacterial feeding RNAi targeting *isp-1* (1/10th strength), *atl-1* (9/10th strength), both genes combined (1/10th strength *isp-1* + 9/10th strength *atl-1*) or vector control, from the L1 larval stage to the first day of adulthood. Animals were collected, washed thrice, then pre-incubated for 30 minutes at RT with rocking to clear the gut of live bacteria, all in S-Basal media. Approximately 30 worms were added to each Seahorse well, with 5 replicate wells employed per test condition. Four test wells were always set aside as worm-free control wells. The Seahorse measurement routine consisted of a 2 minute mix, a 2 minutes wait, then a 2 minute data collection phase, looped 8 times. We followed the procedure of Luz and colleagues [41], and only averaged oxygen consumption rates across the final four measurement phases for each well to provide a single rate value for that well. For each experiment, the 5 biological replicates (wells) were averaged per condition. A total of four independent experiments was performed. Significance testing was undertaken using the Student's t-test, with $p<0.05$ considered significant.

Polysome Profiling. Quantitative polysome profiling was undertaken using the procedure of Steffen and colleagues [42] with the following modifications for *C. elegans:* Synchronized nematode populations (50,000 worms per condition) were cultured on bacterial feeding RNAi targeting *isp-1* (1/10th strength), *atl-1* (9/10th strength), both genes combined (1/10th strength *isp-1* + 9/10th strength *atl-1*) or vector control, from the L1 larval stage to the first day of adulthood. After freshly preparing all solutions on the day of the experiment, worms were washed thrice with S-Basal supplemented with 100 μg/mL cycloheximide. During each wash,

worms were allowed to settle under gravity (~5 mins). Worm samples were next homogenized in Lysis Buffer (40mM Tris-HCl pH 8.0, 235mM KCl, 8mM $MgCl_2$, 0.8 mM EGTA, 160 ng/mL heparin, 160 ng/mL cycloheximide, 1.95mM PMSF, 160 Units RNase inhibitor, 0.8% Triton X-100, 0.08% sodium deoxycholate) using a Balch Homogenizer, as described [88]. Cycloheximide is a translational elongation inhibitor and was added to block ribosomes from running off mRNA transcripts during extraction. Heparin stabilizes mRNA/ribosome complexes. Triton X-100 and sodium deoxycholate were added to collect membrane bound- and cytosolic ribosomes. Equal quantities of whole-worm lysate (900 μL final volume, corresponding to either 1.5 or 2.5 OD_{260} units, depending on the replicate) were loaded onto the top of a 7-47% sucrose gradient then centrifuged in a Beckman SWTi40 rotor at 38000 rpm for 2 hours at 4˚C. Sucrose gradients were prepared directly in centrifuge tubes by layering 5.5 ml 7% sucrose on top of the same volume of 47% sucrose, both prepared in Gradient Buffer (1.6 M KCl, 30mM $MgCl_2$, 100mM Tris-HCl pH 7.5, 1mg/ml heparin, 100 μg/ml cycloheximide). Centrifuge tubes were placed horizontally at RT for 3 hours to allow gradient formation. After loading they were used immediately. Following centrifugation, sample gradients were fractionated using a BR-188 Density Gradient Fractionation System (Brandel), employing 55% sucrose in Gradient Buffer as the chase solution (flow rate: 750 μL/min). Ribosomal RNA-containing elution peaks were monitored via UV absorbance (254 nm) until the entire gradient had been fractionated (~ 30 minutes). Two independent experimental replicates were collected.

Western Analysis. Synchronous populations of SLR001 and SLR004 worms (**Table S4**) were cultured on bacterial feeding RNAi targeting *atp-3* (1/10th strength and undiluted) or vector control, from the L1 larval stage to the first day of adulthood. Unbalanced individuals, representing homozygous *atl-1(tm853)* and wild type worms, respectively, were then manually selected from each plate and whole-worm extracts prepared for western blotting. Briefly, worms were collected and washed thrice in ice-cold S-basal. Next, a cocktail of protease inhibitors in 1.5% SDS (P2714, Sigma Aldrich) was added to the pellet then whole-

worm extracts prepared by boiling for 5 minutes. After centrifugation (17,000xg, 5 mins. 4˚C) the cleared supernatant was retained. For western blotting we used the following reagents and suppliers: 12% NuPAGE gels (Invitrogen); nitrocellulose membrane (Protran, BA83); 5% milk powder in Tris-buffered saline + 0.05% Tween-20 (TBS-$T_{0.05\%}$) for blocking (1 hour, RT) ; TBS-$T_{0.05\%}$ for washing steps, α-ICD-1 primary antibody (1:2000 dilution, [64]) incubated at RT for 2 hours in 5% milk powder in TBS-$T_{0.05\%}$, HRP-coupled goat α-rabbit secondary antibody (ab6721, Abcam) incubated at RT for one hour. α-ICD-1 reactive bands were detected using chemiluminescence (Pierce) and a Typhoon Imaging System (GE Life Sciences).

DNA Damage Response (DDR) Screen.

DDR Feeding RNAi Library Assembly: Bacterial feeding RNAi targeting 201 DNA repair-related genes were sub-cloned from the Ahringer *C. elegans* RNAi Collection (Source Bioscience) into 96 well stock plates. Genes related to DDR repair were identified using WormMine (www.wormbase.org). For details see Torgovnick *et. al*. 2018 [43].

atm-1(gk186); atl-1(tm853) Suppressor Screen: Genes underlying the recovery in sensitivity of unbalanced *atm-1(gk186); atl-1(tm853)* worms (derived from strain SLR005) to *atp-3* RNAi were identified as follows – bacteria expressing feeding RNAi targeting *atp-3* (OD_{590} 0.39) were mixed 1:7 with each of the 201 DDR-related bacterial feeding RNAi clones (each OD_{590} 0.6) then seeded onto 6-cm RNAi plates (NGM agar containing 1 mM IPTG, 100 μg/ml ampicillin, and 5 μg/ml tetracycline; final *atp-3* concentration = 1/10th strength). Lawns were then allowed to mature overnight at room temperature. A parallel set of plates was also prepared except vector-only containing bacteria was used in place of bacteria containing the *atp-3* feeding RNAi construct. Each of the 402 plates was then seeded with 200 arrested L1 larvae (24 hr. arrest) from strain SLR0005 (*atm-1(gk186); atl-1(tm853).* All L1 larvae were the progeny of nT1 parents, meaning they were a mixture of both balanced and unbalanced genotypes. After 72 hours, plates were screened for

unbalanced animals that were differentially recalcitrant to the size reducing effects of *atp-3* RNAi relative to their nT1 siblings. 16 first-round RNAi hits were obtained, which were subsequently clonally re-isolated, sequence-verified, then re-tested using *atp-3* RNAi at 1/10th and ½ strength. Random images of worms from 1/10th strength *atp-3* RNAi plates were collected using an Olympus SZX16 microscope connected to a DP71 CCD camera, then worm lengths were quantified using ImageJ (n= 20 worms per condition, per genotype, per RNAi).

<u>Quantitation of RNAi Effect Size and Significance Testing</u>*:* In order to quantify the degree of recalcitrance to *atp-3* RNAi in the presence of each of the DDR library hits that unbalanced *atm-1(gk186); atl-1(tm853)* worms displayed relative to their nT1-containing (*atm-1(gk186)/+; atl-1(tm853) /+)* siblings, the worm length measurements collected in the previous section were combined with the following *in silico* sampling approach to establish a formal metric. For every test RNAi, an Excel spreadsheet was established that each contained four columns of 100 worm lengths. The worm lengths in each column were randomly chosen from actual worm lengths measured from one of the following categories. Each column corresponded to one sample set.

(i) nT1 worms treated with 9/10th strength test feeding RNAi + 1/10th strength vector feeding RNAi

(ii) nT1 worms treated with 9/10th strength test feeding RNAi + 1/10th strength *atp-3* feeding RNAi

(iii) unbalanced worms treated with 9/10th strength test feeding RNAi + 1/10th strength vector feeding RNAi

(iv) unbalanced worms treated with 9/10th strength test feeding RNAi + 1/10th strength *atp-3* feeding RNAi

The difference in lengths between the unbalanced worms with and without *atp-3* RNAi was divided by the difference in lengths between the nT1worms with and without *atp-3* RNAi, thereby producing a randomly generated population of possible worm length ratios for each test RNAi. The above steps were

repeated five times for each test RNAi to create an average ratio population. Because ratios obtained from normal variables are non-normal, non-parametric techniques using medians and median absolute distance (MAD) in place of mean and standard deviation were employed in tests of significance (**Table S1**, and see [90] for a discussion on robust statistics). Data were then transferred to SPSS (IBM) for significance testing. Using the Kruskal-Wallis Test for all samples, a significant effect of test RNAi on worm length was revealed [**Table S2**, chi-squared(15)=288.36, $p < 0.001$]. Three nonparametric two-sample tests were then used as *post-hoc* tests to compare each test RNAi against vector: Mann-Whitney U Test, Kolmogorov-Smirnov Two-Samples Test, and the Wald-Wolfowitz Runs Test (**Table S3**). Five test RNAi clones yielded significant results for all three post-hoc tests (each $p<0.001$): *vgln-1*, *ubc-1* (independent isolate #2), *rfc-3, scc-3* and Y47D3A.29 (**File S1**). Worms treated with vector in place of test RNAi had a median ratio of 1.61, and a MAD of 0.17. Worms treated with *vgln-1*, *ubc-1* and *rfc-3* had significantly higher ratios than that of the vector (M=1.78, MAD=0.46; M=1.72, MAD=.16; M=1.86, MAD=.35, respectively), while worms treated with *scc-3* and Y47D3A.29 had significantly lower ratios (M=1.44, MAD=.31; M=1.11, MAD=0.37, respectively).

Additional Statistical Testing

Power analysis was undertaken using "PS: Power and Sample Size Calculation" (v3.1.2) [91,92]. For lifespan studies (**Fig. 1C, 1D, 2C**), we used n = (60 – 70) worms per treatment. Assuming a median survival time of 21 days for wild type animals on vector control (growth at 20°C), and a Type I error probability of 0.05, our ability to detect a true 10 day difference in survival between test and control populations (power) was calculated to be (0.568 - 0.634). Sample sizes were restricted from being larger because the rate limiting step in establishing each lifespan study was the need to visually identify, and then manually isolate, unbalanced individuals among 32,000 synchronous F1 progeny. Also, four of the five lines analyzed were

fertile, so *nT1*-containing Po animals also had to be manually isolated prior to collection of their unbalanced F1 progeny, which placed a further constraint on sample size.

For mean difference testing (**Fig. 1B, 1E, 5, 6B-D, 7A, 7B, 7D, S5B-E**) we utilized Student's t-test, unless stated otherwise (**Fig. 3, 4, 7C**). For power calculations, we assumed subject groups were normally distributed with a standard deviation of 0.1, a true difference in experimental and control means of 0.25, and a Type I error rate of 0.05. Under these condition, sample sizes of n = 3 provided 0.607 probability (power) to identify a true difference between means. Probability increased to 0.931 with sample sizes of n = 5. In practice, reagent cost or availability determined the final number of sample replicates that could be collected, and this was usually n = 3.

SUPPLEMENTAL FIGURE LEGENDS

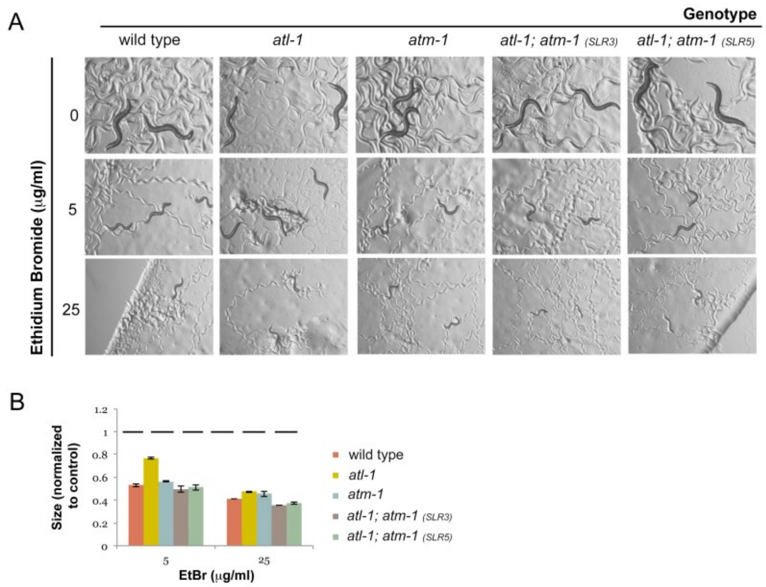

Figure S1. Loss of ATL-1 desensitizes worms to mitochondrial respiratory chain stress induced by ethidium bromide. (**A, B**) Low doses of the potentiometric chemical mutagen ethidium bromide (EtBr) preferentially accumulate in mitochondria, resulting in reduced ETC function as a consequence of disruption to mtDNA integrity. **(A**) Wild type worms cultured from the egg stage to adulthood on either 5 or 25 μg/ml EtBr show a reduction in final adult size. This size reduction is noticeably abrogated by presence of the *atl-1(tm853)* mutation. Representative images were collected randomly. The lengths of the worms shown in each panel are quantified in **(B)**. Bars represent mean +/- range.

A

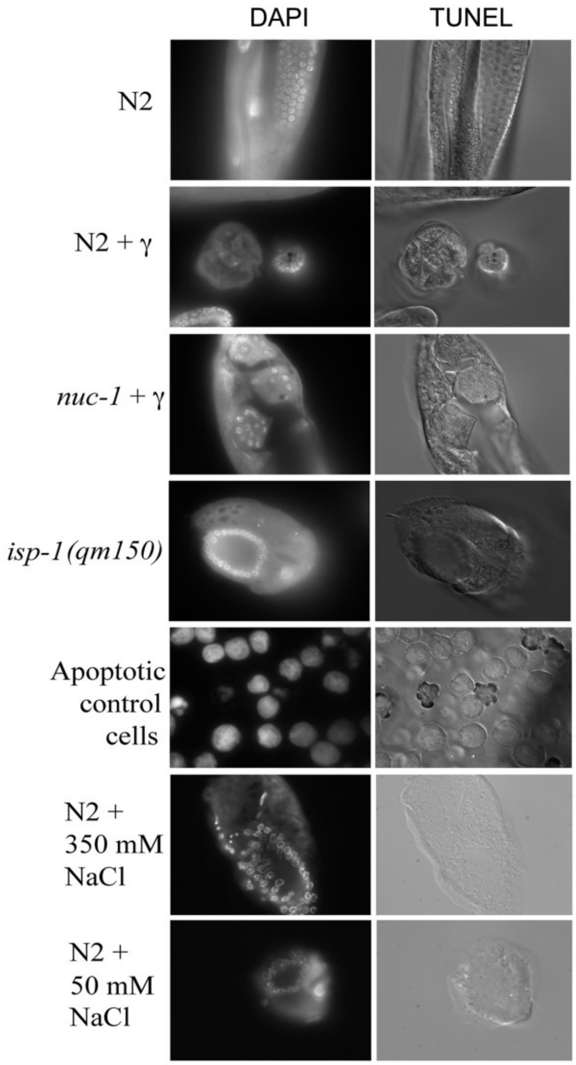
DAPI
TUNEL
N2
N2 + γ
nuc-1 + γ
isp-1(qm150)
Apoptotic control cells
N2 + 350 mM NaCl
N2 + 50 mM NaCl

B

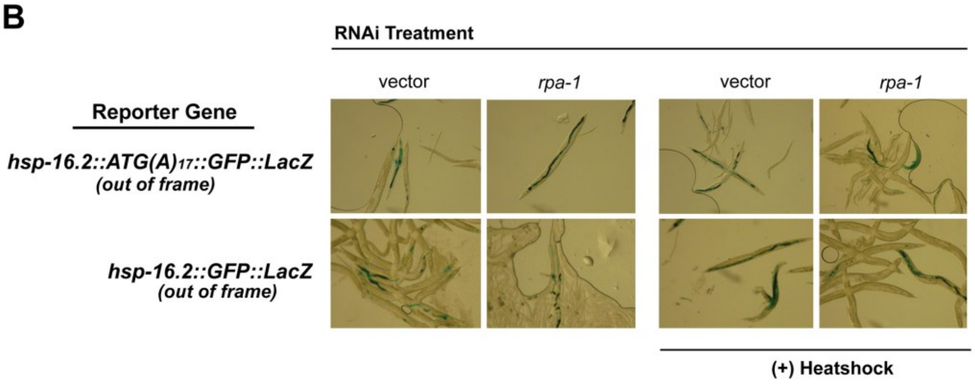
RNAi Treatment
vector
rpa-1
vector
rpa-1
Reporter Gene
hsp-16.2::ATG(A)17::GFP::LacZ (out of frame)
hsp-16.2::GFP::LacZ (out of frame)
(+) Heatshock

Figure S2. DNA mutation detection in *C. elegans* using β-Gal frame-shift- and TUNEL assays. (**A**) No evidence for DNA breaks in *isp-1(qm150)* mutants using TUNEL staining. Strain genotype and treatment condition are listed on *left.* Shown are 3 μM sections of paraffin-embedded worms. Positive controls include wild type (N2) and *nuc-1(e1392)* worms treated with gamma (γ) radiation (1344 Gy), mammalian cells undergoing apoptosis, and worms exposed to osmotic stress (350 mM NaCl). *nuc-1* encodes a DNase II required for DNA degradation during apoptosis. (Note that treatment with 350 mM NaCl was previously reported to increase DNA strand breakage [49], but we could find no support for this result in our studies, despite following the same methodology.) (**B**) P*hsp-16.2::ATG(A)*$_{17}$*::GFP::LacZ* and P*hsp-16.2::ATG::GFP::LacZ* both encode out-of-frame reporter genes [86]. Mutagenic events that bring the reading frame of either reporter back into frame result in a detectable event (LacZ positive staining). Both germline and somatic events are detectable using this approach. HT115 bacteria contain a functional lac operon (blue worms, *vector-only treatment*) that in our hands precludes use of this reporter with the Ahringer RNAi bacterial feeding RNAi library, contrary to previous reports [86].

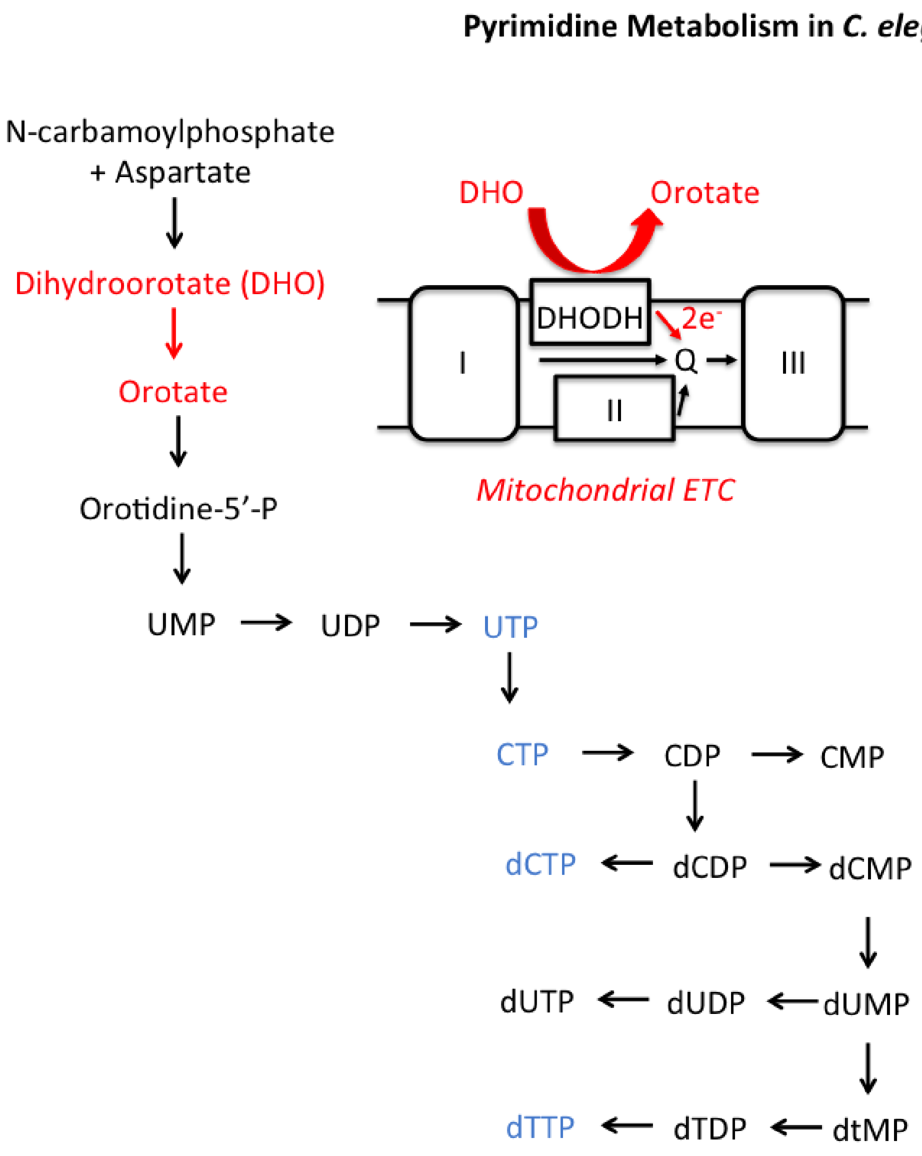

Figure S3. Nucleotide biosynthesis in *C. elegans* highlighting the role of the mitochondrial ETC. Schematic depiction of key intermediates involved in the genesis of pyrimidine nucleotides in worms. The mitochondrial inner membrane-associated enzyme dihydroorotate dehydrogenase (DHODH, named DHOD-1 in *C. elegans*) catalyzes the oxidation of dihydroorotate to orotate and delivers its reducing equivalents directly to ubiquinone in the ETC chain.

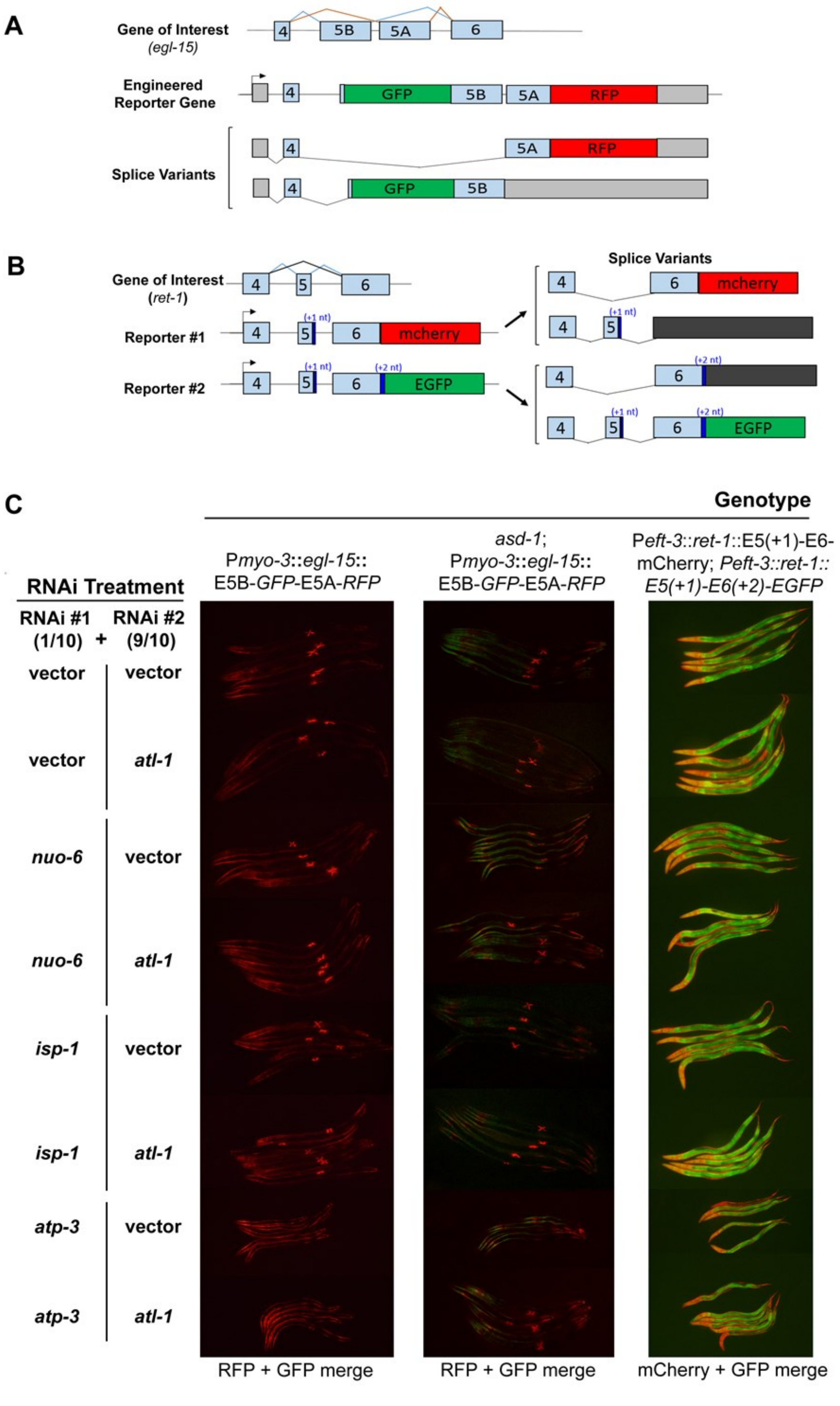

A
Gene of Interest
(egl-15)
4
5B
5A
6
Engineered Reporter Gene
GFP
RFP
Splice Variants
B
Gene of Interest
(ret-1)
Reporter #1
Reporter #2
(+1 nt)
(+2 nt)
mcherry
EGFP
Splice Variants
C
Genotype
Pmyo-3::egl-15::E5B-GFP-E5A-RFP
asd-1; Pmyo-3::egl-15::E5B-GFP-E5A-RFP
Peft-3::ret-1::E5(+1)-E6-mCherry; Peft-3::ret-1::E5(+1)-E6(+2)-EGFP
RNAi Treatment
RNAi #1 (1/10) + RNAi #2 (9/10)
vector
vector
vector
atl-1
nuo-6
vector
nuo-6
atl-1
isp-1
vector
isp-1
atl-1
atp-3
vector
atp-3
atl-1
RFP + GFP merge
RFP + GFP merge
mCherry + GFP merge

Figure S4. mRNA splicing remains unaltered in worms experiencing ETC disruption, both in the absence or presence of *atl-1* knockdown. (**A, B**) Schematic of *egl-15* (A) and *ret-1* (B) splicing-detection reporter gene and gene pair, respectively [39,60]. (**C**) Splicing function reported by the *egl-15 (left panel)* and *ret-1 (right panel)* synthetic reporter constructs remains unaltered by 1/10th strength *atp-3, isp-1* or *nuo-6* knockdown in age-matched worms (vector control are 3rd day adults). *atl-1* knockdown alone, and in combination with 1/10th strength *atp-3, isp-1* or NDUFB4/*nuo-6* RNAi, also did not alter expression of either reporter construct. The *egl-15* reporter gene in the *asd-1(yb978)* mutant background is presented as a positive control (*middle panel*). Wild type ASD-1 binds a region in intron 4 that represses selection of E5B-GFP, thereby enhancing E5A-RFP expression [60].

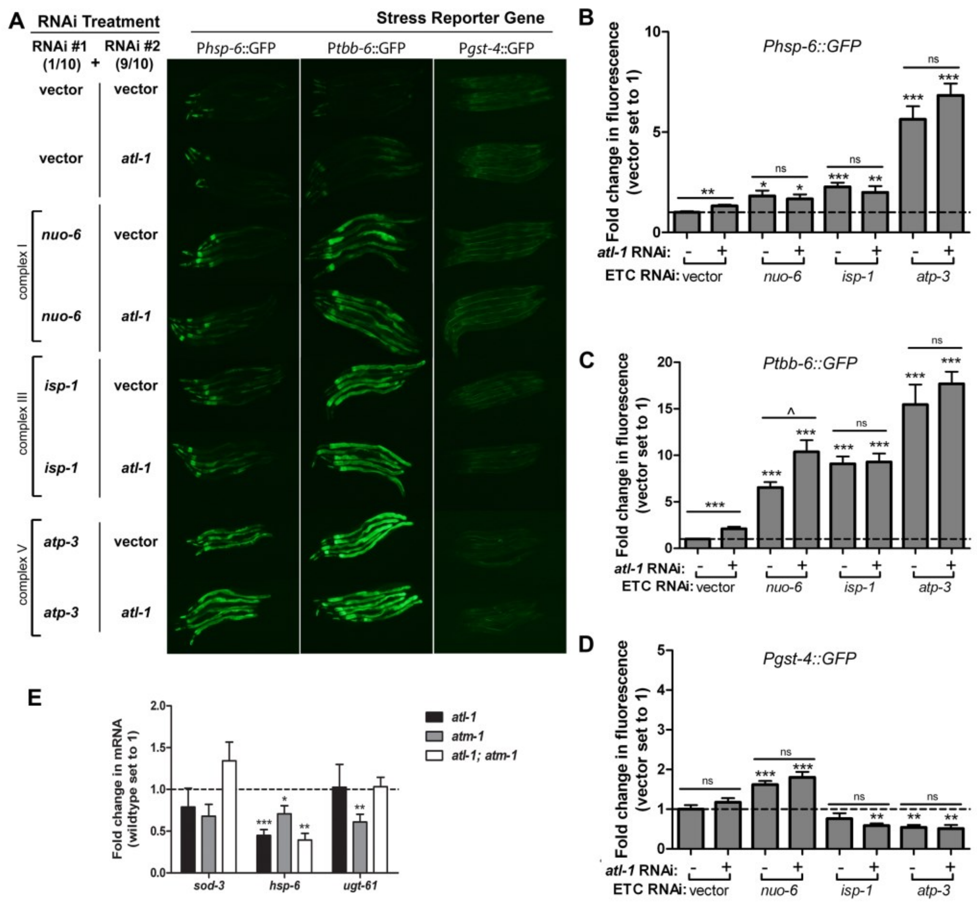

Figure S5. ***atl-1*** **knockdown does not alter retrograde response pathways normally induced by mitochondrial ETC stress.** (**A-D**) Reporter genes monitoring the mitochondrial unfolded protein response (*Phsp-6::GFP),* the PMK-3/p38-dependent retrograde response (*Ptbb-6::GFP*) and the SKN-1 dependent oxidative stress response (*Pgst-4::GFP*) following *atp-3, isp-1* and *nuo-6* knockdown (A). Quantification provided in *panels* (B-D). *atl-1* knockdown does not cause hyperactivation of any reporter gene, except *Ptbb-6::GFP,* which is mildly (1.6 fold) increased in *nuo-6* RNAi-treated worms. (Asterisks indicate significantly different from vector unless otherwise noted by bar over comparison group: *Student's t-test,* * p<0.05, **p<0.01, ***p<0.001, ^ p < 0.01; error bars: SEM.) (**E**) Abundance of endogenous *sod-3, hsp-6* and *ugt-61* mRNA transcripts in *atl-1(tn853)* mutants relative to wild type worms (data for each mRNA is normalized

to wild type). Transcript abundance in *atm-1(gk186)* and *atl-1(tm853); atm-1(gk186)* (line SLR0003) worms is also provided. All animals are the unbalanced F1 progeny of parents carrying the nT1 reciprocal chromosomal translocation, including wild type control. (Asterisks indicate significantly different from wild type: *Student's t-test*, * $p<0.05$, **$p<0.01$, ***$p<0.001$, Error bars: SEM.)

SUPPLEMENTAL TABLE LEGENDS

Table S1. Summary of descriptive statistics for final-round DDR test RNAi screen hits. (Related to **Fig. 3.**)

Test RNAi	Median	MAD	Variance	Skewness	Kurtosis	Min	Max
Vector	1.61	0.17	0.25	1.92	7.81	1.18	3.00
crn-1	1.84	0.28	0.17	0.67	0.16	1.11	3.16
hpr-17	1.69	0.34	7.57	7.78	71.75	-3.67	27.44
ung-1 (isolate 1)	1.70	0.24	0.14	0.77	1.28	0.98	3.07
ung-1 (isolate 2)	1.55	0.21	0.07	1.03	1.98	1.08	2.54
C08H9.2	1.94	0.26	1.03	4.77	27.71	1.36	8.99
ubc-1 (isolate 1)	1.72	0.16	0.05	0.34	-0.46	1.33	2.35
ubc-1 (isolate 2)	1.81	0.21	0.11	0.91	0.79	1.31	3.09
ubc-1 (isolate 3)	1.71	0.30	0.16	2.73	11.40	1.15	3.94
rfc-3	1.86	0.35	2.37	-4.14	27.15	-8.50	5.57
lin-40	1.70	0.18	0.07	0.85	1.92	1.10	2.83
gsp-1	1.57	0.27	0.18	0.85	0.49	0.83	2.97
scc-3	1.44	0.31	0.30	0.40	-0.18	0.38	2.88
sir-2.2	1.67	0.21	0.13	1.42	4.05	1.17	3.48
Y47D3A.29	1.22	0.21	0.12	1.00	1.02	0.73	2.52
ape-1	1.86	0.21	0.25	3.87	23.55	1.31	5.41

Note. N=100 for all groups.

Table S2. Mean Ranks of final-round DDR RNAi hits used for Kruskal-Wallis test. (Related to **Fig. 3.**)

Test RNAi	N	Mean Rank
Vector	100	692.54
crn-1	100	969.4
hpr-17	100	837.69
ung-1 (isolate 1)	100	788.82
ung-1 (isolate 2)	100	581.33
C08H9.2	100	1130.45*
ubc-1 (isolate 1)	100	833.1
ubc-1 (isolate 2)	100	998.47*
ubc-1 (isolate 3)	100	836.1
rfc-3	100	989.36*
lin-40	100	833.11
gsp-1	100	693.31
scc-3	100	522.83*
sir-2.2	100	825.33
Y47D3A.29	100	324.45*
ape-1	100	951.71

** test RNAi is significantly different from the vector, as revealed in post-hoc analysis*

Table S3. ***Post-hoc*** **tests for each final-round, DDR RNAi hit versus vector.** (Related to **Fig. 3.**)

Test RNAi	Significance Test[a]	Significance	Retain or Reject Null Hypothesis?
crn-1	Mann – Whitney U Test	.000	Reject*
	Kolmogorov – Smirnov Test	.000	Reject*
	Wald – Wolfowitz Runs Test	.024	Retain
hpr-17	Mann – Whitney U Test	.159	Retain
	Kolmogorov – Smirnov Test	.000	Reject*
	Wald – Wolfowitz Runs Test	.024	Retain
ung-1 (isolate 1)	Mann – Whitney U Test	.136	Retain
	Kolmogorov – Smirnov Test	.002	Retain
	Wald – Wolfowitz Runs Test	.000	Reject*
ung-1 (isolate 2)	Mann – Whitney U Test	.011	Retain
	Kolmogorov – Smirnov Test	.024	Retain
	Wald – Wolfowitz Runs Test	.008	Retain
C08H9.2	Mann – Whitney U Test	.000	Reject*
	Kolmogorov – Smirnov Test	.000	Reject*
	Wald – Wolfowitz Runs Test	.000	Reject*
ubc-1 (isolate 1)	Mann – Whitney U Test	.003	Retain
	Kolmogorov – Smirnov Test	.002	Retain
	Wald – Wolfowitz Runs Test	.012	Retain
ubc-1 (isolate 2)	Mann – Whitney U Test	.000	Reject*

	Kolmogorov – Smirnov Test	.000	Reject*
	Wald – Wolfowitz Runs Test	.001	Reject*
ubc-1 (isolate 3)	Mann – Whitney U Test	.004	Retain
	Kolmogorov – Smirnov Test	.010	Retain
	Wald – Wolfowitz Runs Test	.024	Retain
rfc-3	Mann – Whitney U Test	.000	Reject*
	Kolmogorov – Smirnov Test	.000	Reject*
	Wald – Wolfowitz Runs Test	.000	Reject*
lin-40	Mann – Whitney U Test	.005	Retain
	Kolmogorov – Smirnov Test	.010	Retain
	Wald – Wolfowitz Runs Test	.033	Retain
gsp-1	Mann – Whitney U Test	.471	Retain
	Kolmogorov – Smirnov Test	.006	Retain
	Wald – Wolfowitz Runs Test	.004	Retain
scc-3	Mann – Whitney U Test	.000	Reject*
	Kolmogorov – Smirnov Test	.000	Reject*
	Wald – Wolfowitz Runs Test	.000	Reject*
sir-2.2	Mann – Whitney U Test	.025	Retain
	Kolmogorov – Smirnov Test	.006	Retain
	Wald – Wolfowitz Runs Test	.197	Retain
Y47D3A.29	Mann – Whitney U Test	.000	Reject*

	Kolmogorov – Smirnov Test	.000	Reject*
	Wald – Wolfowitz Runs Test	.000	Reject*
ape-1	Mann – Whitney U Test	.000	Reject*
	Kolmogorov – Smirnov Test	.000	Reject*
	Wald – Wolfowitz Runs Test	.012	Retain

Note. *p<.001

[a]These are nonparametric significance tests comparing each test RNAi to the vector.

Table S4. List of *C. elegans* strains employed in current study

Strain	*Genotype**	Source	Strain Construction**
BC2200	*dpy-18(e364)/eT1 III; unc-46(e177)/eT1 V*	CGC	
CB1392	*nuc-1(e1392) X*	CGC	
CB3388	*ncl-1(e1865) III*	CGC	
CB665	*unc-58(e665) X*	CGC	
CF2218	*ncl-1(e1942) III*	CGC	
CL2166	*dvIs19[pAF15(Pgst-4::GFP::NLS)] III*	CGC	
DW101 ***	*atl-1(tm853) /nT1 (qIs50) (IV;V)*	Simon J. Boulton (London Res. Instit.)	
KH1125	*asd-1 (yb978); ybIs733 [myo-3::egl-15::BGAR + lin-15(+)]*	Hidehito Kuroyanagi (TMDU)	*Kuroyanagi, H., et al. Nature Methods. 2007*
KH2283	*lin-15 (n765) ybIs2167 [eft-3::ret-1E4E5(+1)E6-GGS6-mCherry + eft-3::ret-1E4E5(+1)E6-(+2)GGS6-EGFP + lin-15 (+) + pRG5271Neo] X (outcrossed 7x)*	Hidehito Kuroyanagi (TMDU)	*Kuroyanagi, H., et al. Nucleic Acids Res. 2013*
KH928	*ybIs733 [myo-3::egl-15::BGAR + lin-15 (+)] (outcrossed 7x)*	Hidehito Kuroyanagi (TMDU)	*Kuroyanagi, H., et al. Nature Methods. 2006*
N2	*wild-type*	CGC	
NL3400	*pkIs1604 [hsp-16.2::ATG(A)17GFP::LacZ + rol-6(su1006)]*	CGC	
NL3401	*pkIs1605 [hsp-16.2p::GFP::LacZ + rol-6(su1006)]*	CGC	
SJ4100	*cIs13[Phsp-6::GFP] V*	CGC	
SLR0001	*atl-1 (tm853) / nT1 (qIs50) (IV;V)*	Rea Lab	VC381 x DW101
SLR0002	*atm-1(gk186) I; + / nT1 (qIs50) (IV;V)*	Rea Lab	VC381 x DW101
SLR0003	*atm-1(gk186) I; atl-1 (tm853) / nT1 (qIs50) (IV;V)*	Rea Lab	VC381 x DW101
SLR0004	*+/ nT1 (qIs50) (IV;V)*	Rea Lab	VC381 x DW101
SLR0005	*atm-1(gk186) I; atl-1 (tm853) / nT1 (qIs50) (IV;V)*	Rea Lab	VC381 x DW101

SLR0008	*+/nT1(IV);+/nT1(V)*	Rea Lab	TJ5026 x DW101
SLR0011	*+/nT1(IV);+/nT1(V)*	Rea Lab	TJ5026 x DW101
SLR0012	*+/nT(IV);atl-1(tm853)/nt1(V)*	Rea Lab	TJ5026 x DW101
SLR0014	*+/nT(IV);atl-1(tm853)/nt1(V)*	Rea Lab	TJ5026 x DW101
SLR0016	*isp-1(qm150)(IV)/ nT1 (qIS50)(IV);+/nT1(V)*	Rea Lab	TJ5026 x DW101
SLR0019	*gst-4::gfp(III); isp1(qm150)/nT1(qIs50)(IV);+/nT1(V)*	Rea Lab	TJ5026 x DW101
SLR0020	*isp-1(qm150)/nT1(qIs50)(IV);atl-1(tm853)/nT1(V)*	Rea Lab	TJ5026 x DW101
SLR0023	*isp-1(qm150)/nT1(qIs50)(IV);atl-1(tm853)/nT1(V)*	Rea Lab	TJ5026 x DW101
SLR0115	*dvIs67[pCL179(Ptbb-6::GFP) + Pmyo-3::dsRed]*	Rea Lab	*Munkacsy E, et. al. PLoS Genet. 2016;12(7):e1006133*
SP457	*unc-93(e1500) III*	CGC	
TJ376	*(N2:zExH25P06.1::GFP)*	Thomas E. Johnson (UC Boulder)	*Henderson, S., et al. J Geron. 2006*
TJ564	*isp-1(qm150) IV; dvIs19[pAF15(Pgst-4::GFP::NLS)] III*	Rea Lab	
TJ5013	*+ / nT1[unc^{d}(n754); let](IV;V)*	Rea Lab	
TJ5026	*gst-4::gfp(III); isp1(qm150)/ nT1[unc^{d}(n754); let](IV;V)*	Rea Lab	TJ564 x DW101
VC381	*atm-1(gk186) I*	CGC	

*For all transcriptional reporters, genes contributing the promoter are prefixed with P

**For crosses, the hermaphrodite strain is written first, followed by the male

*** *qIs50* encodes a pharangeal GFP marker. Between the pair of nT1 chromosomes, it is integrated on the one that balances *isp-1(qm150) IV*

Table S5. qPCR primer list.

Gene	Forward Primer (5'-3')	Reverse Primer (5'-3')	Citation
cdc-42	CTGCTGGACAGGAAGATTACG	CTCGGACATTCTCGAATGAAG	Hoogewijs, *et al.* 2008 BMC Mol Bio
pmp-3	GTTCCCGTGTTCATCACTCAT	ACACCGTCGAGAAGCTGTAGA	Hoogewijs, *et al.* 2008 BMC Mol Bio
Y45F10D.4	GTCCTTCAAATCAGTTCAGC	GTTCTTGTCAAGTGATCCGAC	Hoogewijs, *et al.* 2008 BMC Mol Bio
atp-3	ACGGTGACTTATGCCGTCAAG	TTAGATGGCGGTGGCAAGG	Rea, *et al*. 2007 PLOS Biology
ama-1 intron 4	GAATAAAAAGTGGTTTTAAGCCCG	CAAATTTTCCTAAACCGCAATTGTG	
ctb-1 (mtDNA)	CGCCCGATAGGTTAATAGCA	TGGCCCCATTAAAATGAAAA	Sugimoto, *et al.* 2008 Exp Cell Res
nuo-6	ATCTCCGAAACTGTGGCAG	TCTTCAAAATCTCCTTGACACG	
mev-1	AGGATTCGATTTGGCTAAGGG	CAAGAGTTGAAGACAATGGCG	
isp-1	GATGCTGGAGATTACGGAGG	ATCCTTGAACGAGTATGCTGG	
cco-1	CGCTACCGGACGTGAAAAG	GCATCCAATGATTCTGAAGTCG	
atl-1	CCAAACCTGTAATCATTAAACTCACG	GTGTCTGCTCGTCCTTCATTAG	
sod-3	TCAAAGGAGCTGATGGACA	CAATATCCCAACCATCCCC	
hsp-6	GATTGGATAAGGACGCTGGAG	GAGGAATGTATCTCCGTTGGTG	
ugt-61	AGAAGGATCTACTGTTGCACG	GGTTTCTTGGCGATTTGGGAG	

OTHER SUPPLEMENTAL FILES

File S2. Mean difference ratios of all hits from DNA Damage Response (DDR) screen. (Related to **Fig. 3.**)

File S3. Significance testing for differences in HXK2::GFP fluorescence. (Related to **Fig. 7C.**)
