# Supplementary material for: Inhibition of ATR Reverses a Mitochondrial Respiratory Insufficiency
Source: Cells. 2022 May 24;11(11):1731. doi: 10.3390/cells11111731 (PMC9179431; doi:10.3390/cells11111731)
Supplement: Supplementary file 1 [file cells-11-01731-s001.zip › cells-1721133-proofed-supplementary/File S2.pdf]

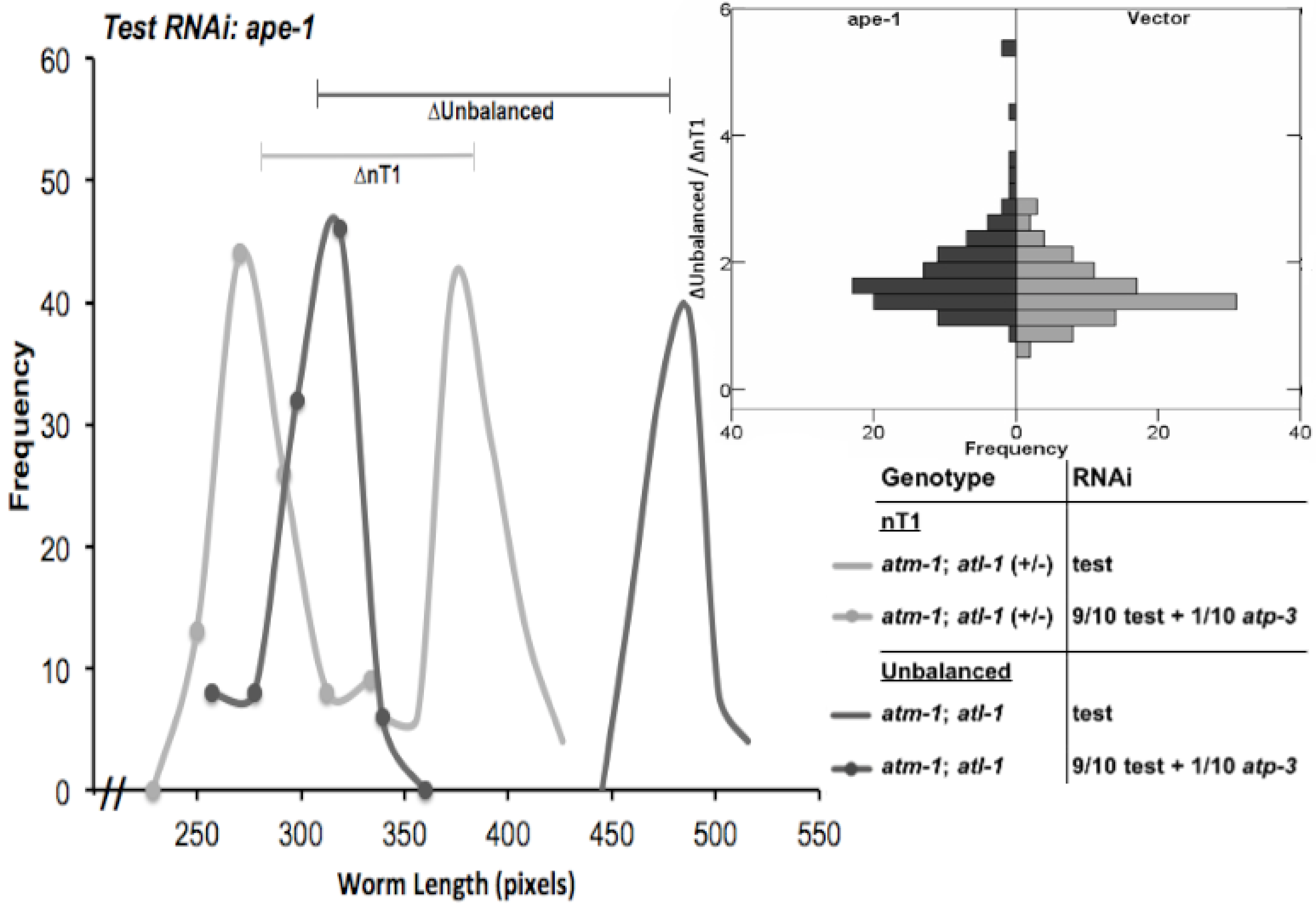

Test RNAi: ape-1
ΔUnbalanced
ΔnT1
Frequency
Worm Length (pixels)
60
50
40
30
20
10
0
250
300
350
400
450
500
550
ape-1
Vector
ΔUnbalanced / ΔnT1
Frequency
6
4
2
0
40
20
0
20
40
Genotype
RNAi
nT1
atm-1; atl-1 (+/-)
test
atm-1; atl-1 (+/-)
9/10 test + 1/10 atp-3
Unbalanced
atm-1; atl-1
test
atm-1; atl-1
9/10 test + 1/10 atp-3

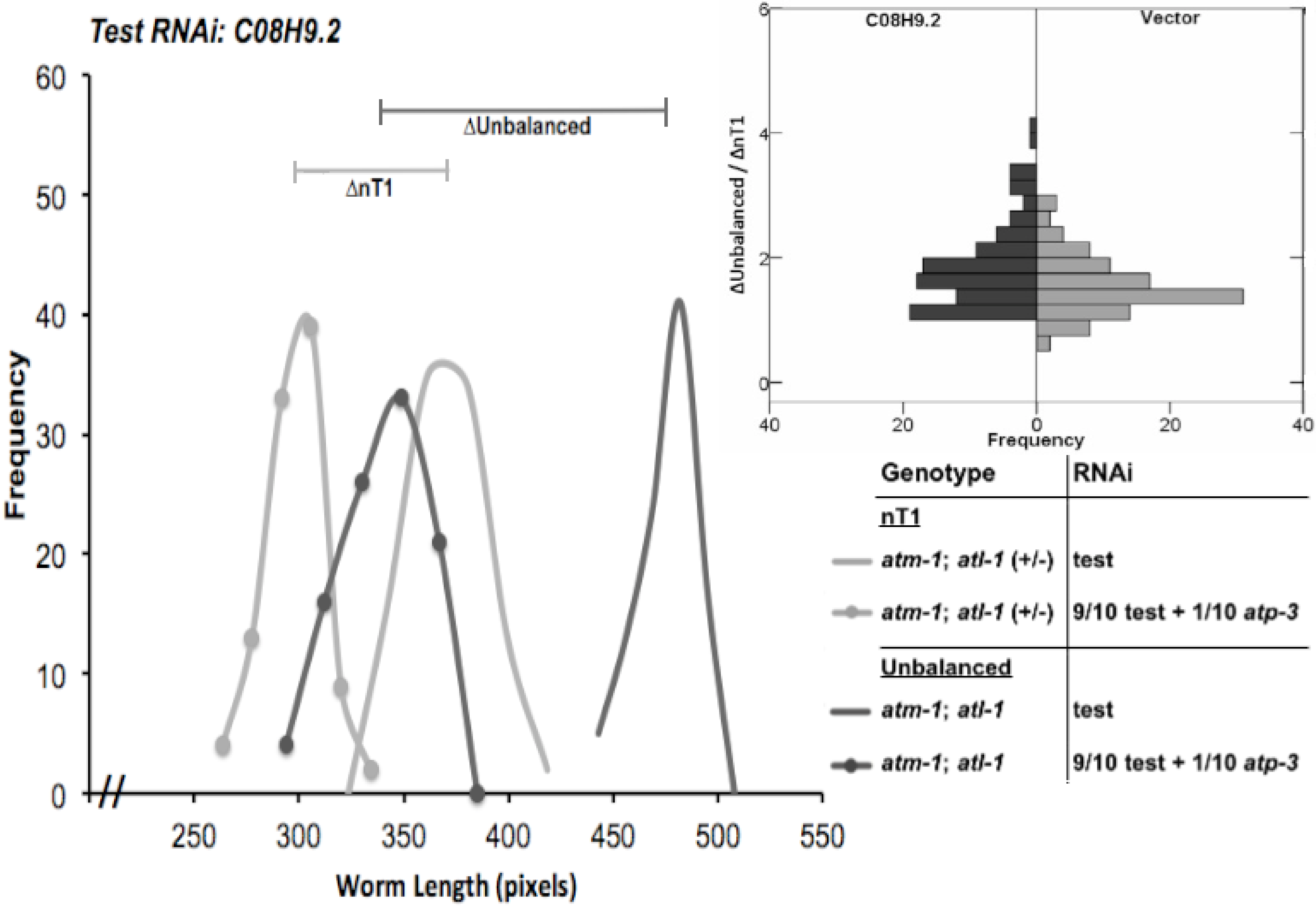

Test RNAi: C08H9.2
Frequency
Worm Length (pixels)
ΔUnbalanced
ΔnT1
C08H9.2
Vector
ΔUnbalanced / ΔnT1
Frequency
Genotype
RNAi
nT1
atm-1; atl-1 (+/-)
test
atm-1; atl-1 (+/-)
9/10 test + 1/10 atp-3
Unbalanced
atm-1; atl-1
test
atm-1; atl-1
9/10 test + 1/10 atp-3

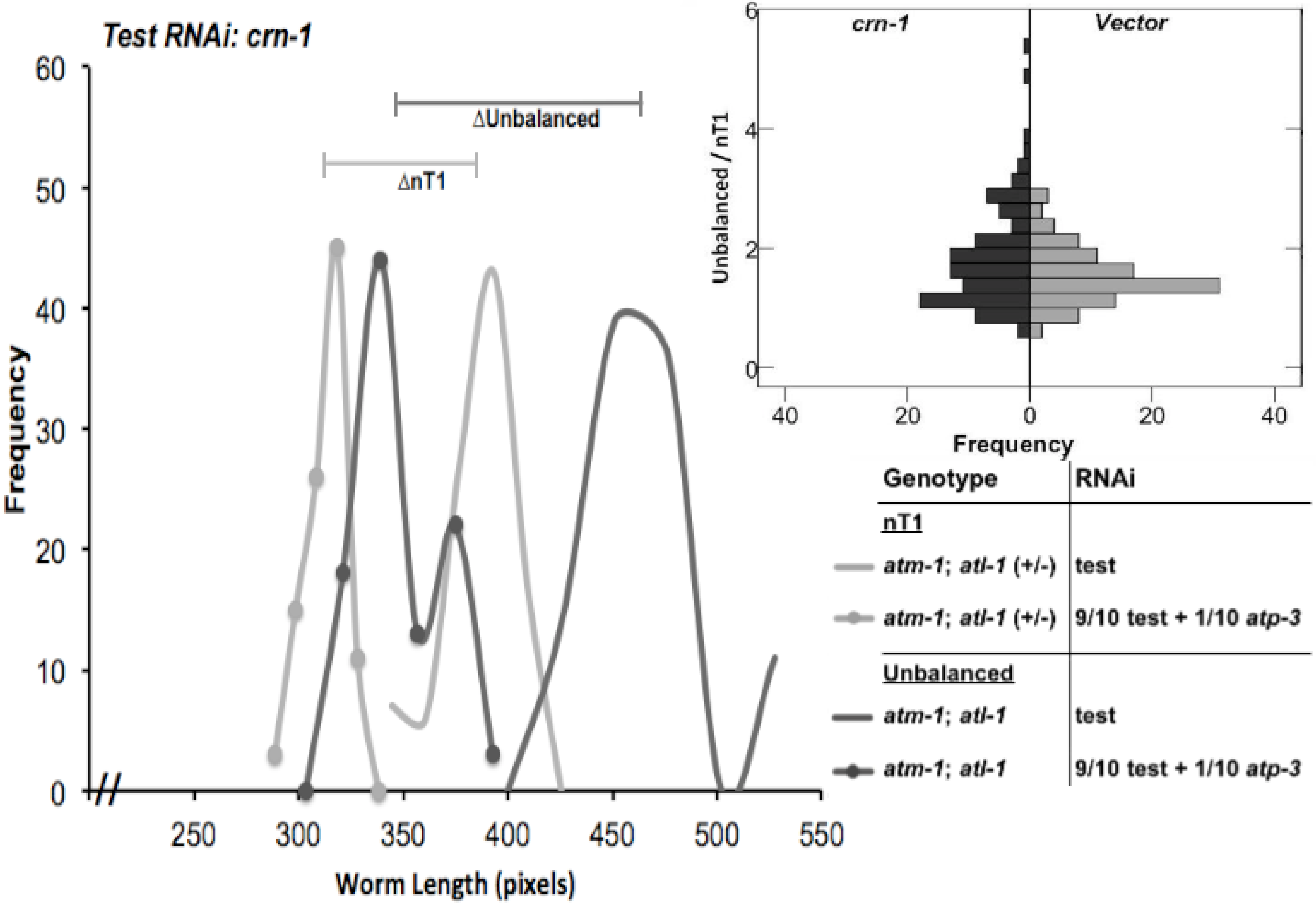

Test RNAi: crn-1
ΔUnbalanced
ΔnT1
Frequency
Worm Length (pixels)
60
50
40
30
20
10
0
250
300
350
400
450
500
550
crn-1
Vector
Unbalanced / nT1
Frequency
6
4
2
0
40
20
0
20
40
Genotype
RNAi
nT1
atm-1; atl-1 (+/-)
test
atm-1; atl-1 (+/-)
9/10 test + 1/10 atp-3
Unbalanced
atm-1; atl-1
test
atm-1; atl-1
9/10 test + 1/10 atp-3

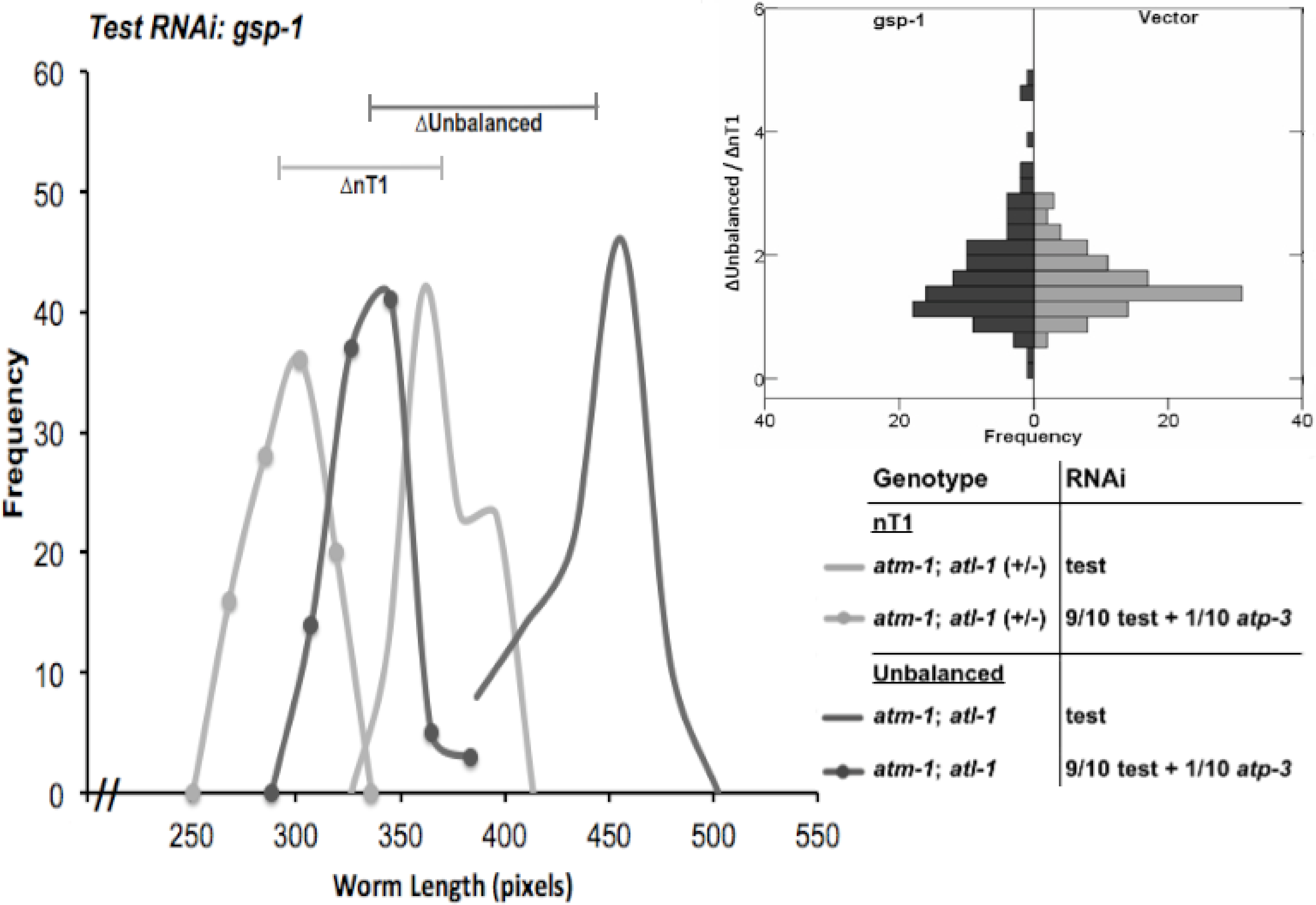

Test RNAi: gsp-1
Frequency
Worm Length (pixels)
ΔUnbalanced
ΔnT1
0
10
20
30
40
50
60
250
300
350
400
450
500
550
gsp-1
Vector
ΔUnbalanced / ΔnT1
Frequency
Genotype
RNAi
nT1
atm-1; atl-1 (+/-)
test
atm-1; atl-1 (+/-)
9/10 test + 1/10 atp-3
Unbalanced
atm-1; atl-1
test
atm-1; atl-1
9/10 test + 1/10 atp-3

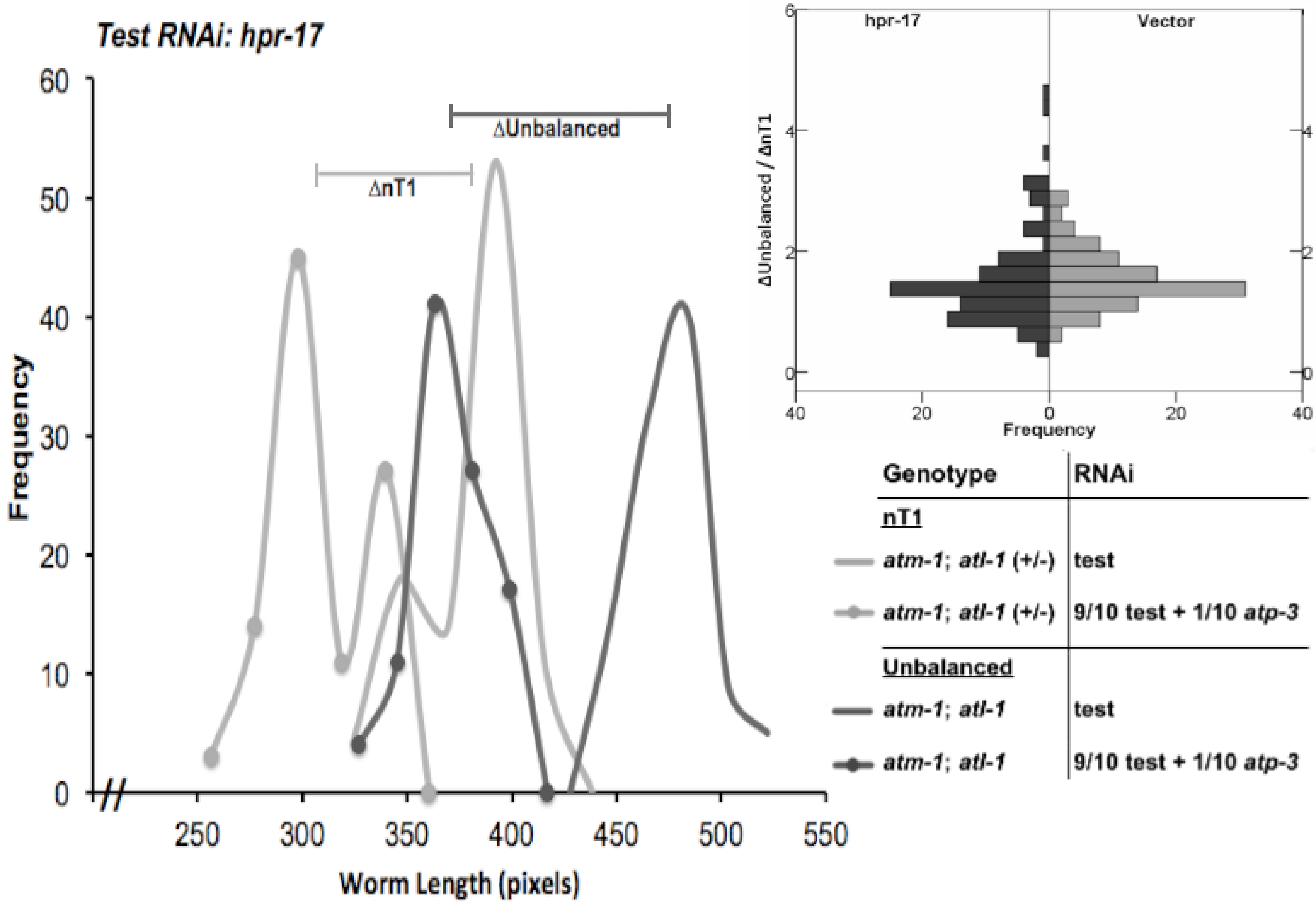
Test RNAi: hpr-17
Frequency
Worm Length (pixels)
ΔnT1
ΔUnbalanced
0
10
20
30
40
50
60
250
300
350
400
450
500
550
hpr-17
Vector
ΔUnbalanced / ΔnT1
Frequency
40
20
0
20
40
0
2
4
6
Genotype
RNAi
nT1
atm-1; atl-1 (+/-)
test
atm-1; atl-1 (+/-)
9/10 test + 1/10 atp-3
Unbalanced
atm-1; atl-1
test
atm-1; atl-1
9/10 test + 1/10 atp-3

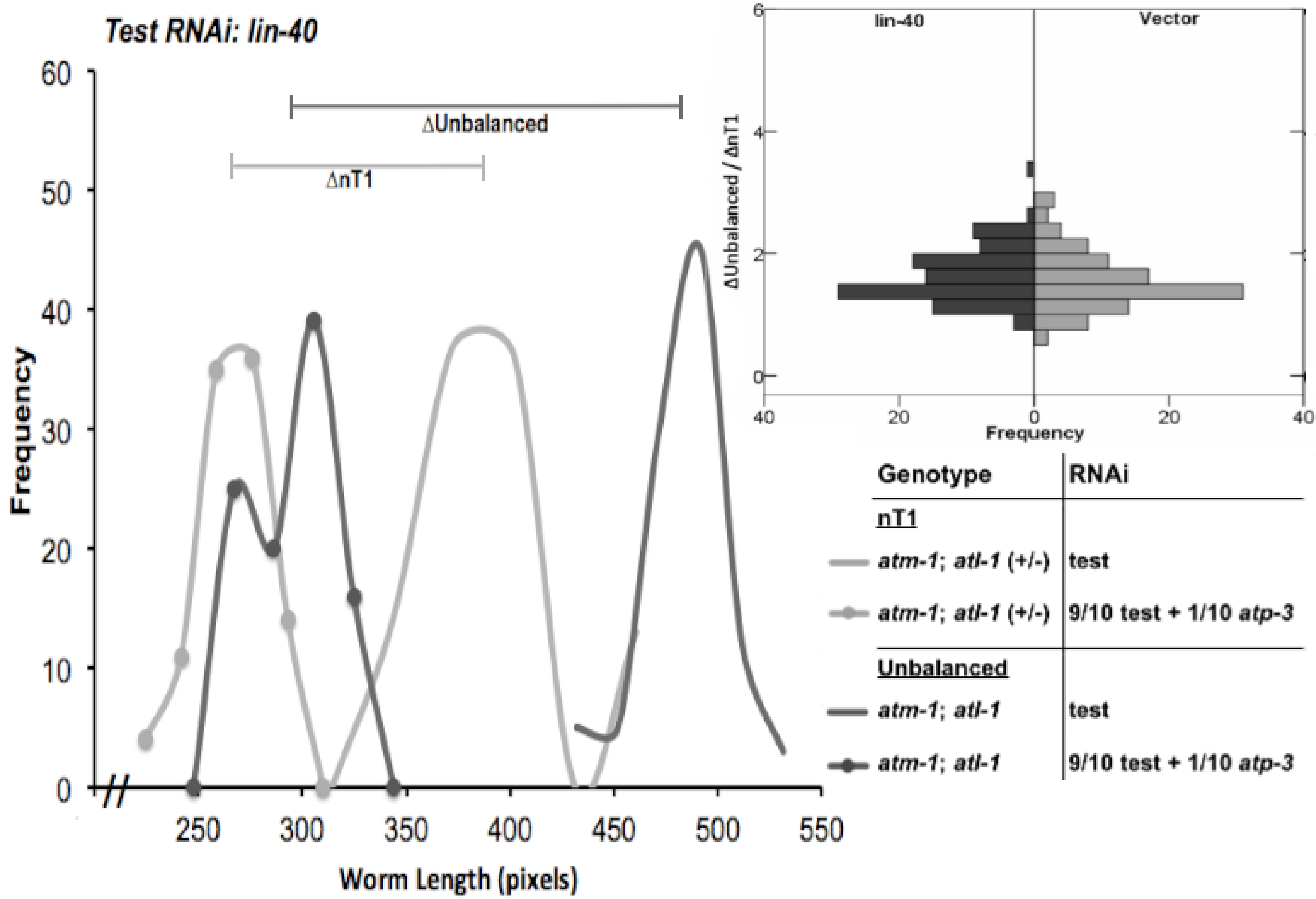
Test RNAi: lin-40
ΔUnbalanced
ΔnT1
Frequency
Worm Length (pixels)
0
10
20
30
40
50
60
250
300
350
400
450
500
550
lin-40
Vector
ΔUnbalanced / ΔnT1
Frequency
0
2
4
6
40
20
0
20
40
Genotype
RNAi
nT1
atm-1; atl-1 (+/-)
test
atm-1; atl-1 (+/-)
9/10 test + 1/10 atp-3
Unbalanced
atm-1; atl-1
test
atm-1; atl-1
9/10 test + 1/10 atp-3

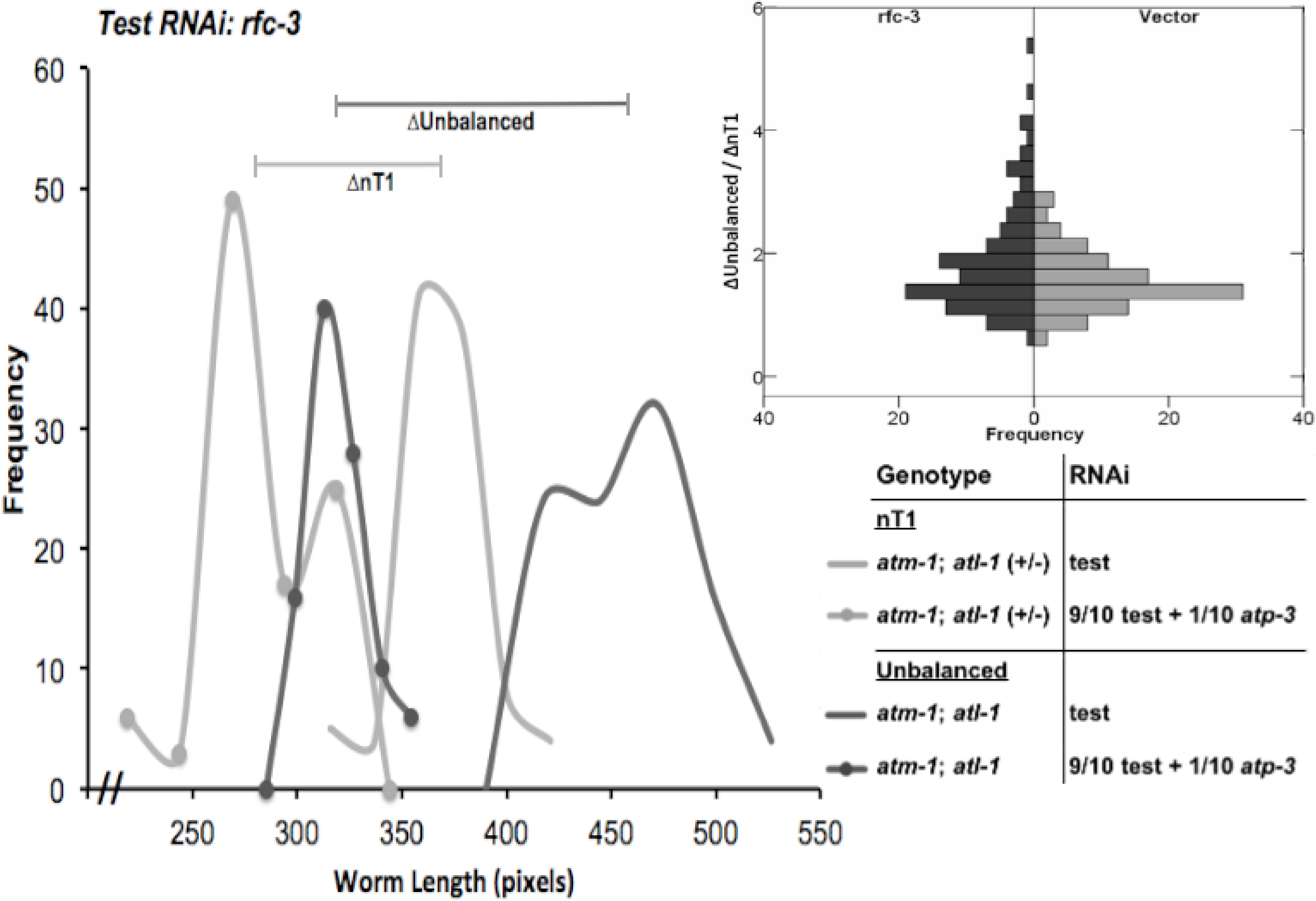

Test RNAi: rfc-3
Frequency
Worm Length (pixels)
ΔUnbalanced
ΔnT1
60
50
40
30
20
10
0
250
300
350
400
450
500
550
rfc-3
Vector
ΔUnbalanced / ΔnT1
Frequency
6
4
2
0
40
20
0
20
40
Genotype
RNAi
nT1
atm-1; atl-1 (+/-)
test
atm-1; atl-1 (+/-)
9/10 test + 1/10 atp-3
Unbalanced
atm-1; atl-1
test
atm-1; atl-1
9/10 test + 1/10 atp-3

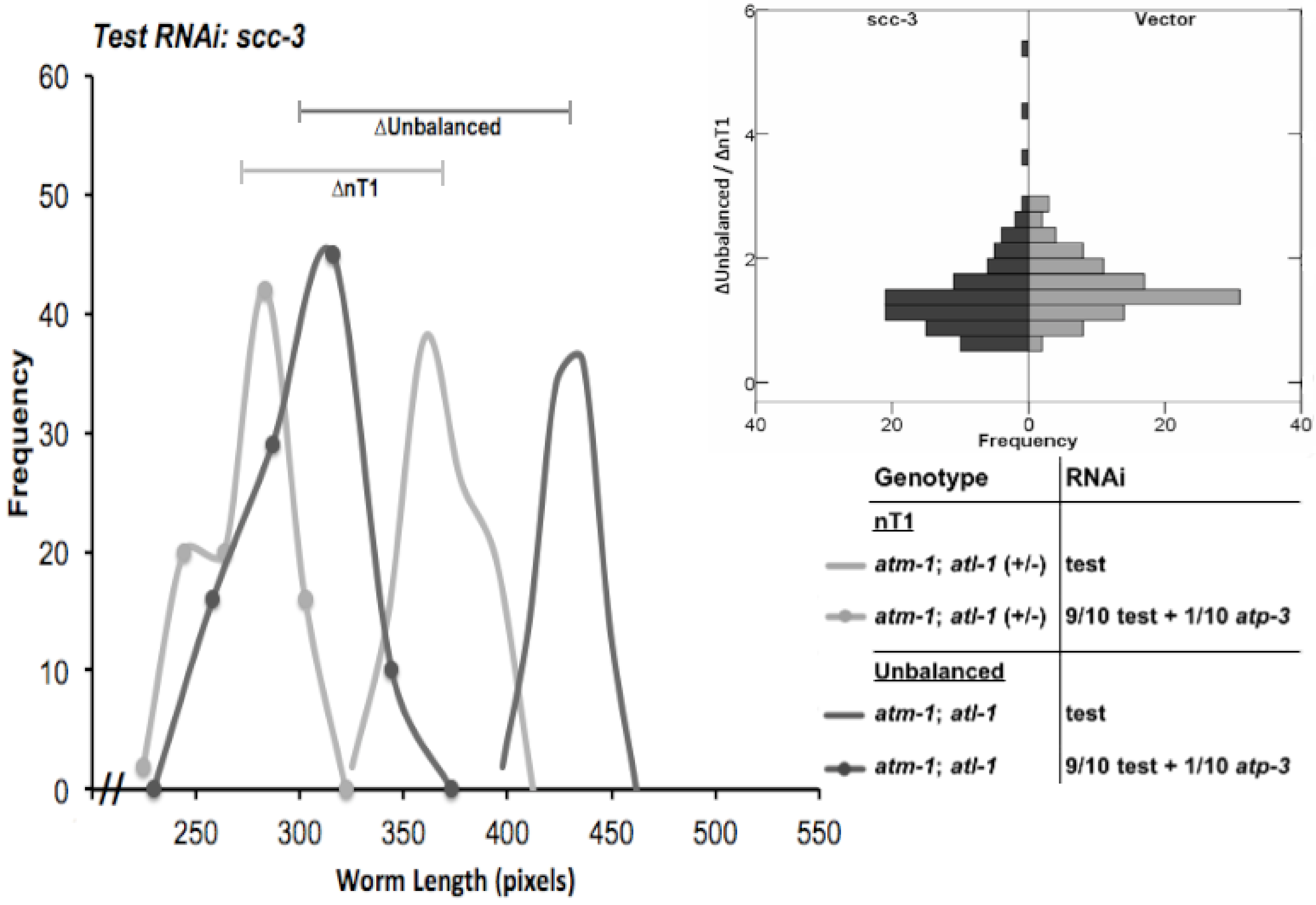

Test RNAi: scc-3
ΔUnbalanced
ΔnT1
Frequency
Worm Length (pixels)
0
10
20
30
40
50
60
250
300
350
400
450
500
550
scc-3
Vector
ΔUnbalanced / ΔnT1
Frequency
0
2
4
6
40
20
0
20
40
Genotype
RNAi
nT1
atm-1; atl-1 (+/-)
test
atm-1; atl-1 (+/-)
9/10 test + 1/10 atp-3
Unbalanced
atm-1; atl-1
test
atm-1; atl-1
9/10 test + 1/10 atp-3

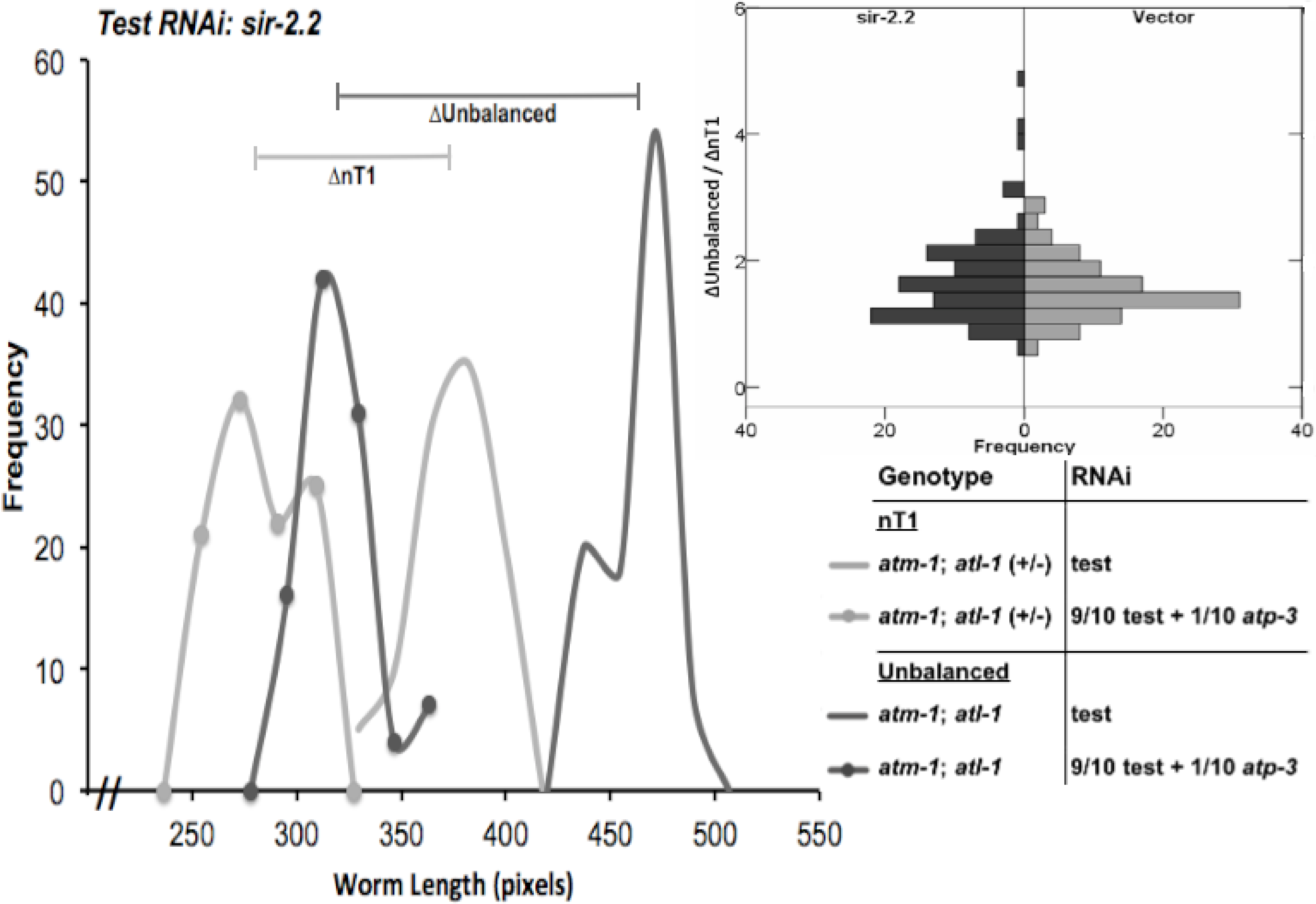

Test RNAi: sir-2.2
ΔUnbalanced
ΔnT1
Frequency
Worm Length (pixels)
60
50
40
30
20
10
0
250
300
350
400
450
500
550
sir-2.2
Vector
ΔUnbalanced / ΔnT1
6
4
2
0
40
20
0
20
40
Frequency
Genotype
RNAi
nT1
atm-1; atl-1 (+/-)
test
atm-1; atl-1 (+/-)
9/10 test + 1/10 atp-3
Unbalanced
atm-1; atl-1
test
atm-1; atl-1
9/10 test + 1/10 atp-3

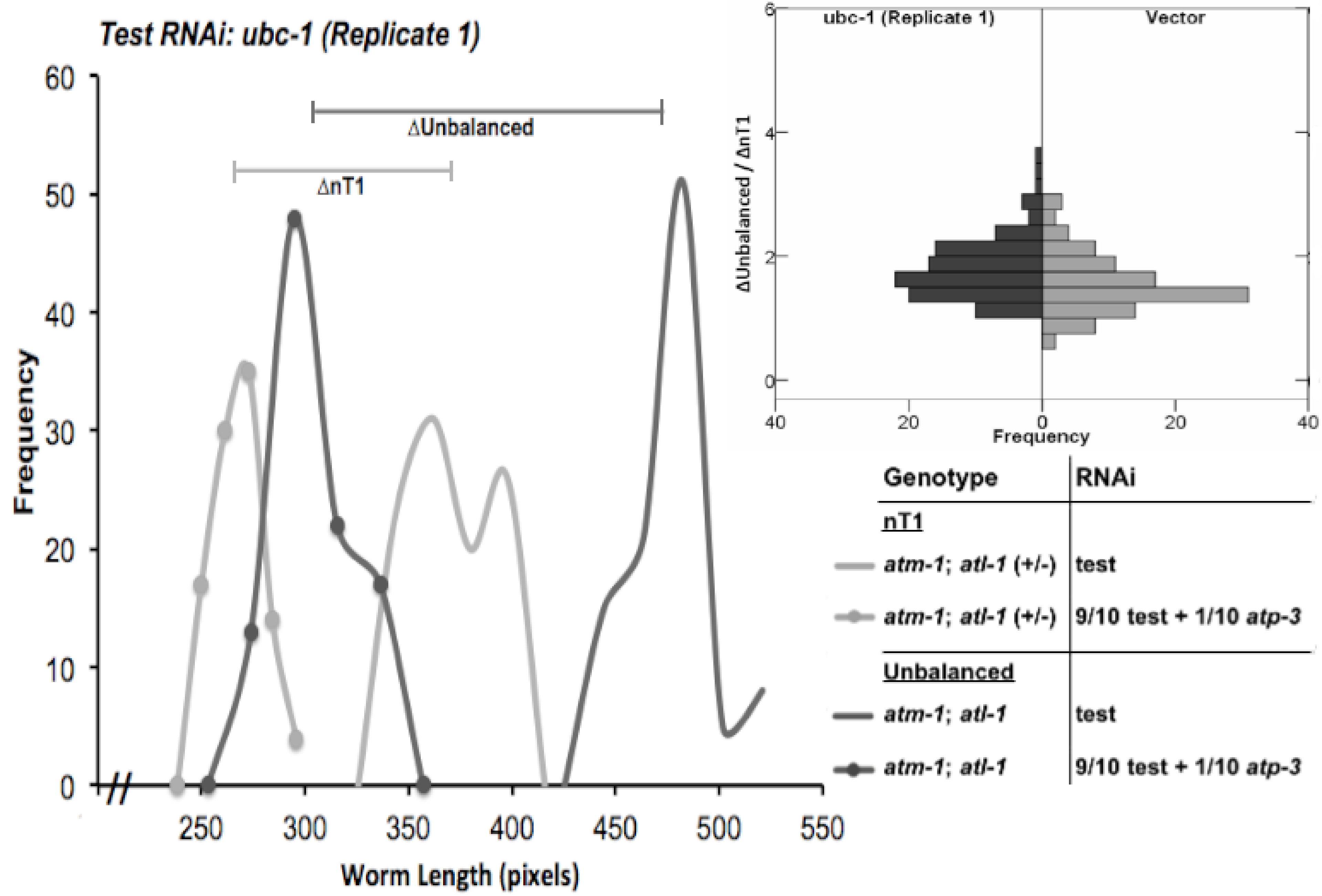
Test RNAi: ubc-1 (Replicate 1)
Frequency
Worm Length (pixels)
ΔUnbalanced
ΔnT1
ubc-1 (Replicate 1)
Vector
ΔUnbalanced / ΔnT1
Frequency
Genotype
RNAi
nT1
atm-1; atl-1 (+/-)
test
atm-1; atl-1 (+/-)
9/10 test + 1/10 atp-3
Unbalanced
atm-1; atl-1
test
atm-1; atl-1
9/10 test + 1/10 atp-3

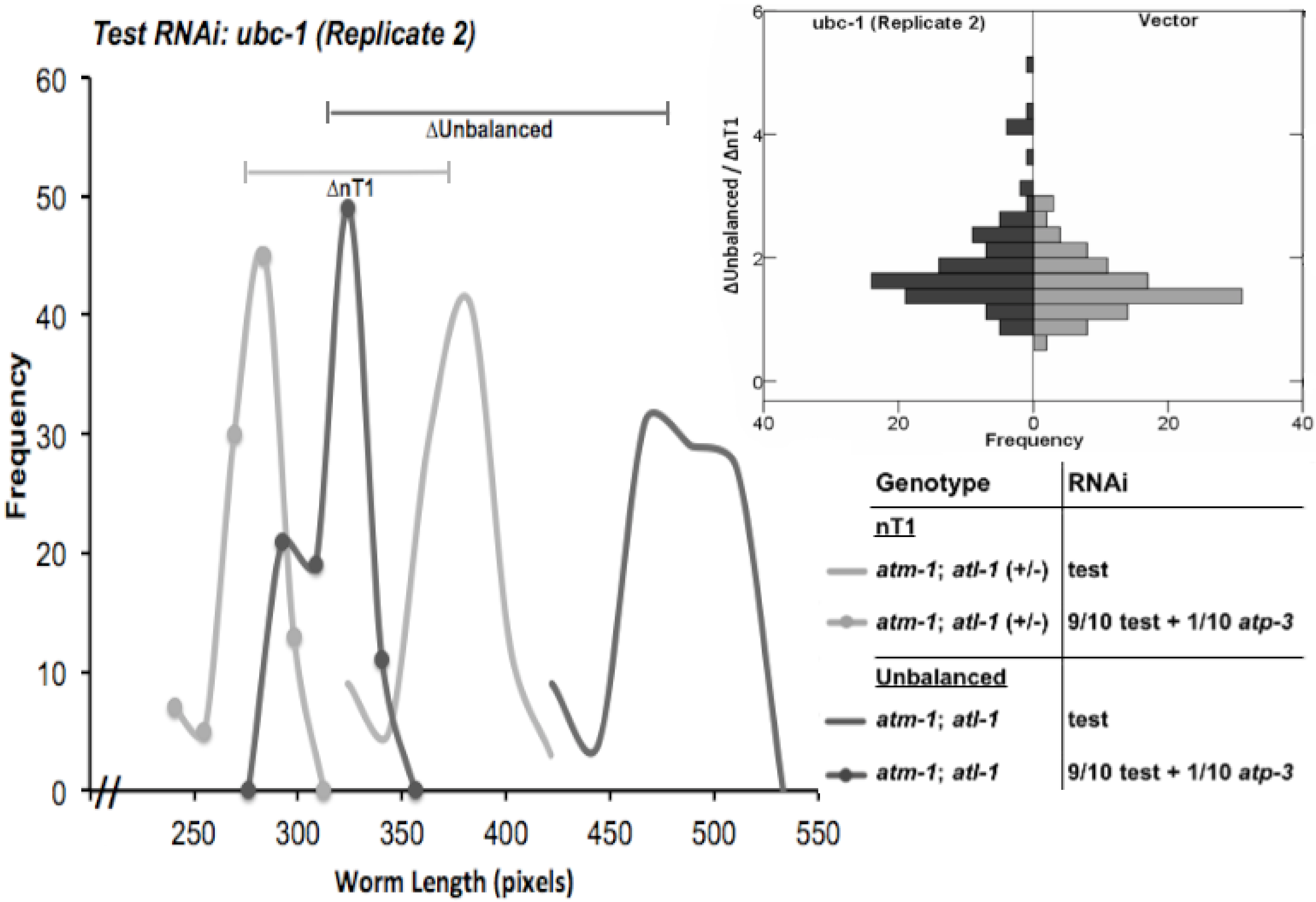
Test RNAi: ubc-1 (Replicate 2)
ΔUnbalanced
ΔnT1
Frequency
Worm Length (pixels)
0
10
20
30
40
50
60
250
300
350
400
450
500
550
ubc-1 (Replicate 2)
Vector
ΔUnbalanced / ΔnT1
Frequency
0
2
4
6
40
20
0
20
40
Genotype
RNAi
nT1
atm-1; atl-1 (+/-)
test
atm-1; atl-1 (+/-)
9/10 test + 1/10 atp-3
Unbalanced
atm-1; atl-1
test
atm-1; atl-1
9/10 test + 1/10 atp-3

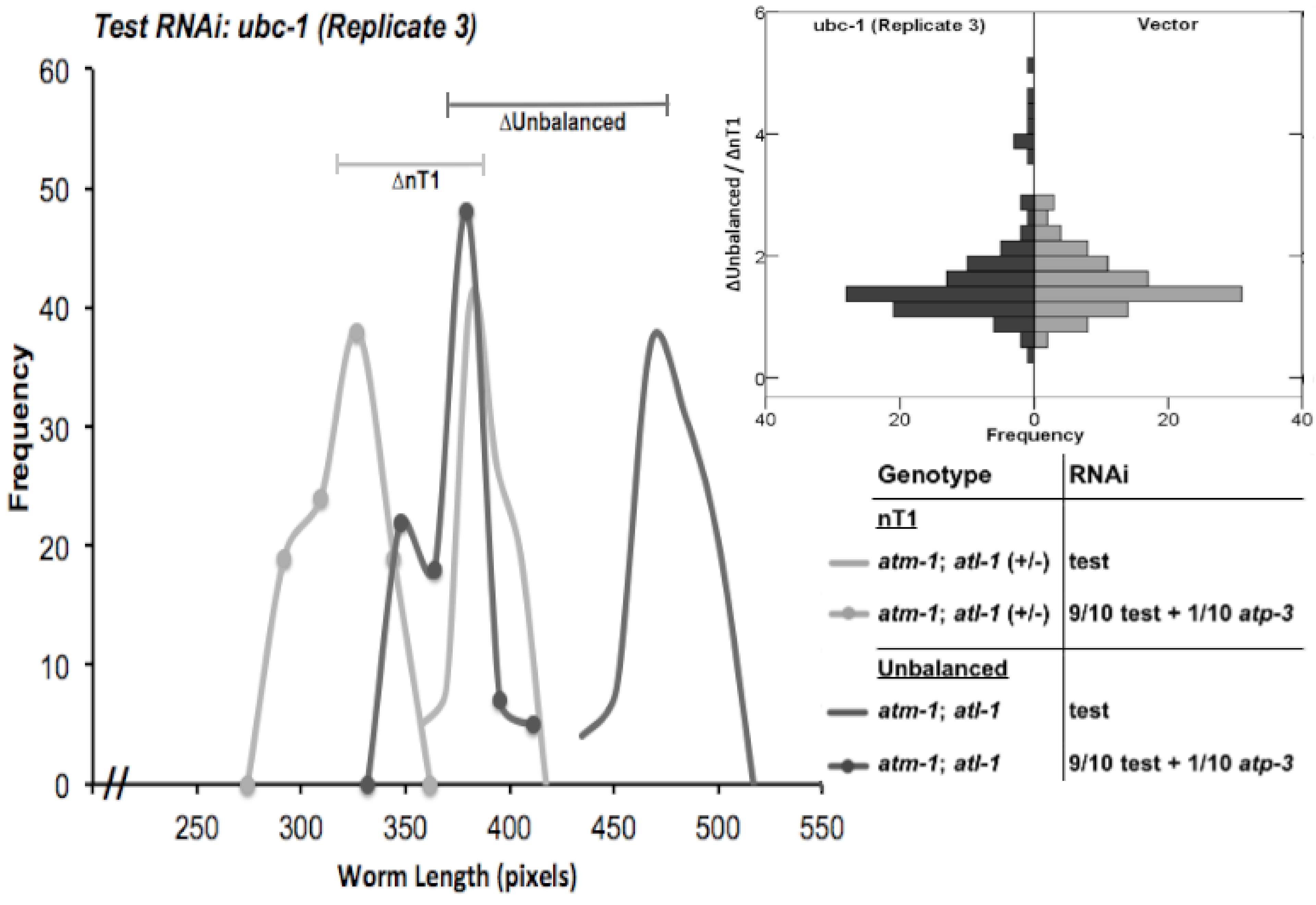

Test RNAi: ubc-1 (Replicate 3)
ΔUnbalanced
ΔnT1
Frequency
Worm Length (pixels)
0
10
20
30
40
50
60
250
300
350
400
450
500
550
ubc-1 (Replicate 3)
Vector
ΔUnbalanced / ΔnT1
Frequency
40
20
0
20
40
0
2
4
6
Genotype
RNAi
nT1
atm-1; atl-1 (+/-)
test
atm-1; atl-1 (+/-)
9/10 test + 1/10 atp-3
Unbalanced
atm-1; atl-1
test
atm-1; atl-1
9/10 test + 1/10 atp-3

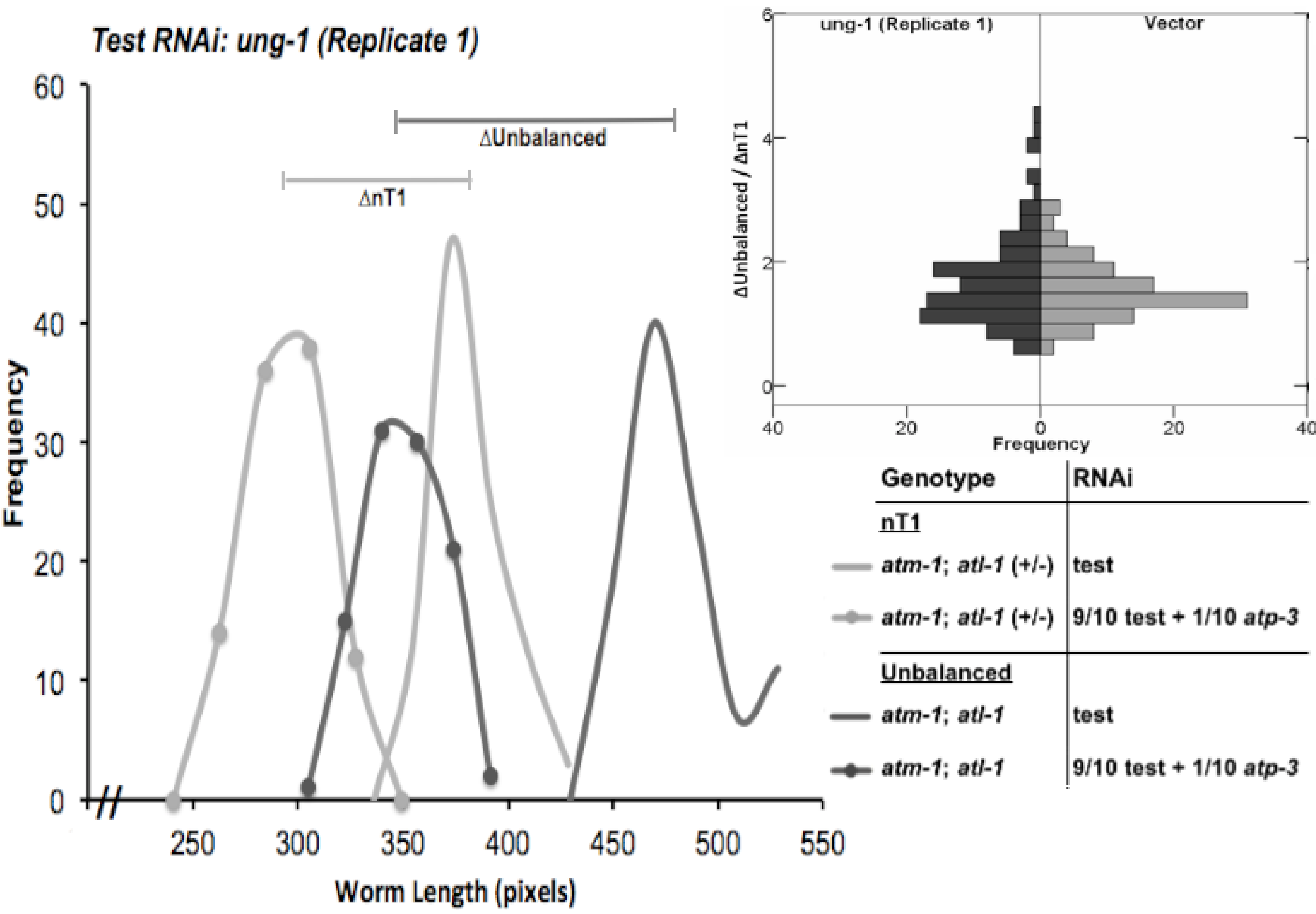

Test RNAi: ung-1 (Replicate 1)
Frequency
Worm Length (pixels)
ΔUnbalanced
ΔnT1
60
50
40
30
20
10
0
250
300
350
400
450
500
550
ung-1 (Replicate 1)
Vector
ΔUnbalanced / ΔnT1
Frequency
6
4
2
0
40
20
0
20
40
Genotype
RNAi
nT1
atm-1; atl-1 (+/-)
test
atm-1; atl-1 (+/-)
9/10 test + 1/10 atp-3
Unbalanced
atm-1; atl-1
test
atm-1; atl-1
9/10 test + 1/10 atp-3

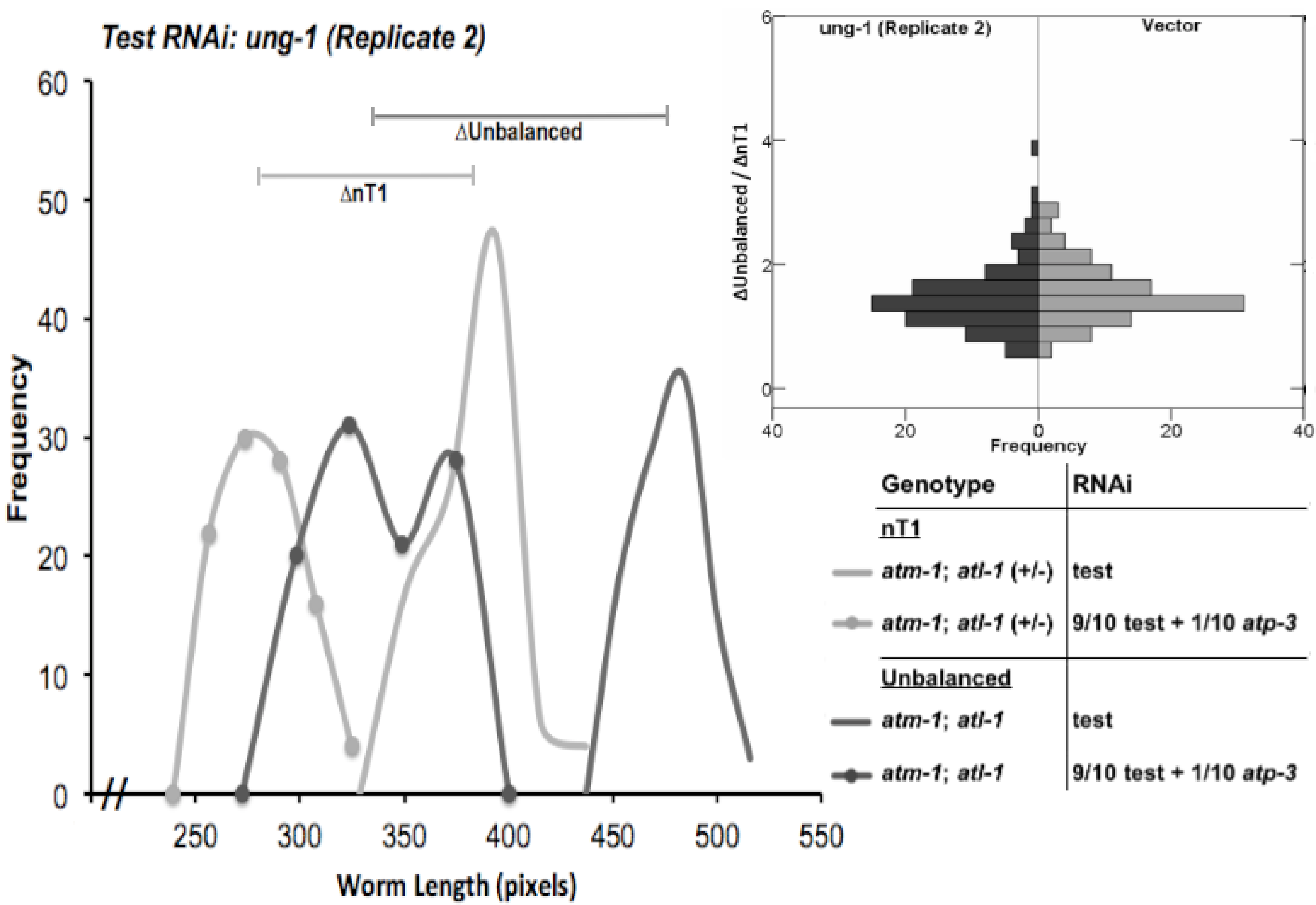
Test RNAi: ung-1 (Replicate 2)
Frequency
Worm Length (pixels)
ΔUnbalanced
ΔnT1
0
10
20
30
40
50
60
250
300
350
400
450
500
550
ung-1 (Replicate 2)
Vector
ΔUnbalanced / ΔnT1
Frequency
0
2
4
6
40
20
0
20
40
Genotype
RNAi
nT1
atm-1; atl-1 (+/-)
test
atm-1; atl-1 (+/-)
9/10 test + 1/10 atp-3
Unbalanced
atm-1; atl-1
test
atm-1; atl-1
9/10 test + 1/10 atp-3

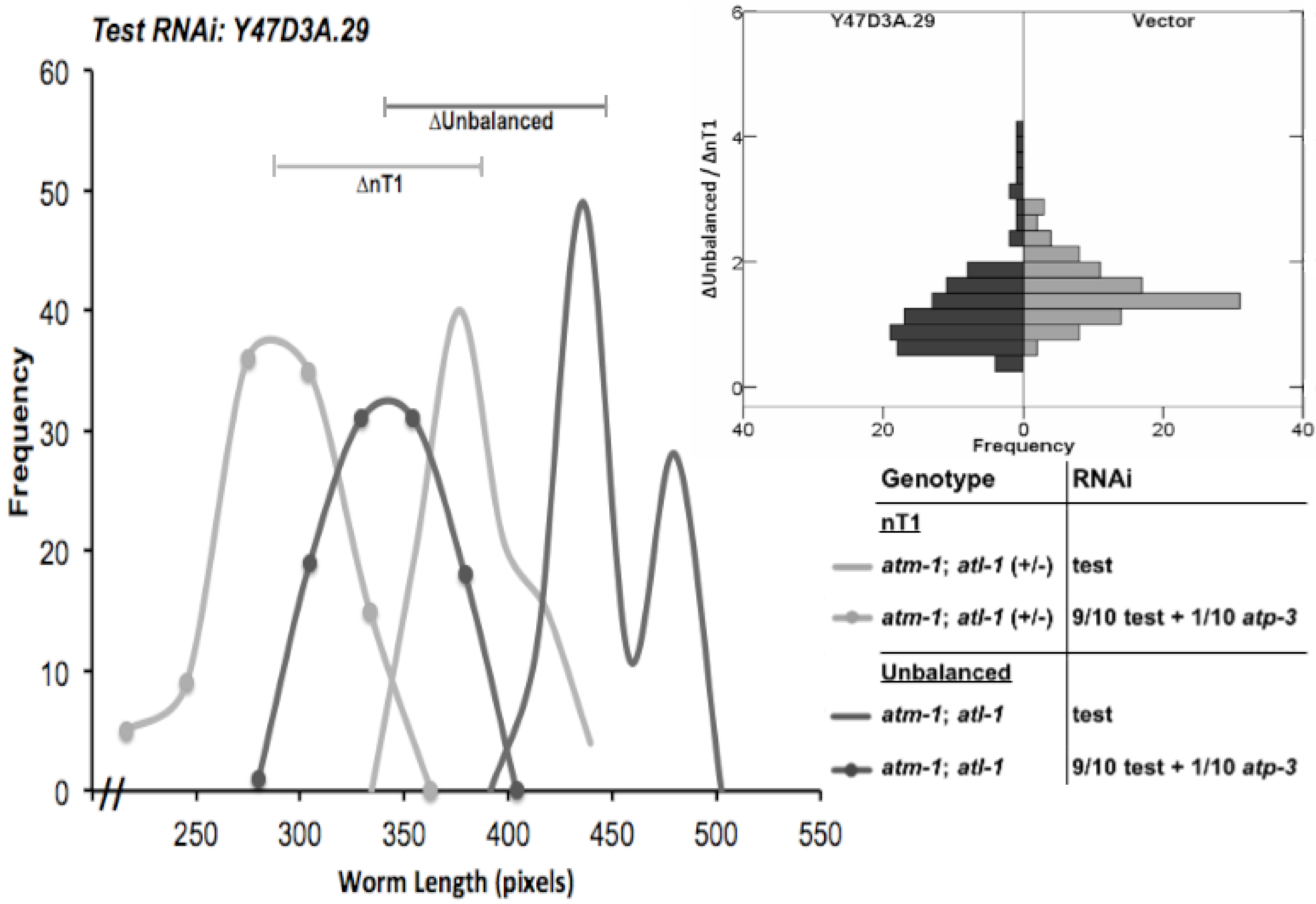

Test RNAi: Y47D3A.29
ΔUnbalanced
ΔnT1
Frequency
Worm Length (pixels)
Y47D3A.29
Vector
ΔUnbalanced / ΔnT1
Frequency
Genotype
RNAi
nT1
atm-1; atl-1 (+/-)
test
atm-1; atl-1 (+/-)
9/10 test + 1/10 atp-3
Unbalanced
atm-1; atl-1
test
atm-1; atl-1
9/10 test + 1/10 atp-3
